# The multiple evolutionary origins of the eukaryotic *N-*glycosylation pathway

**DOI:** 10.1186/s13062-016-0137-2

**Published:** 2016-08-04

**Authors:** Jonathan Lombard

**Affiliations:** 1 National Evolutionary Synthesis Center, 2024 W. Main Street Suite A200, Durham, NC 27705 USA; 2 Biosciences, University of Exeter, Geoffrey Pope Building, Stocker Road, Exeter, EX4 4QD UK

**Keywords:** *N-*glycosylation, Eukaryotes, Archaea, Bacteria, Eukaryogenesis, Prokaryotic cell walls, Polyisoprenol, Glycosyltransferase

## Abstract

**Background:**

The *N-*glycosylation is an essential protein modification taking place in the membranes of the endoplasmic reticulum (ER) in eukaryotes and the plasma membranes in archaea. It shares mechanistic similarities based on the use of polyisoprenol lipid carriers with other glycosylation pathways involved in the synthesis of bacterial cell wall components (e.g. peptidoglycan and teichoic acids). Here, a phylogenomic analysis was carried out to examine the validity of rival hypotheses suggesting alternative archaeal or bacterial origins to the eukaryotic *N-*glycosylation pathway.

**Results:**

The comparison of several polyisoprenol-based glycosylation pathways from the three domains of life shows that most of the implicated proteins belong to a limited number of superfamilies. The *N-*glycosylation pathway enzymes are ancestral to the eukaryotes, but their origins are mixed: Alg7, Dpm and maybe also one gene of the glycosyltransferase 1 (GT1) superfamily and Stt3 have proteoarchaeal (TACK superphylum) origins; *alg2/alg11* may have resulted from the duplication of the original GT1 gene; the lumen glycosyltransferases were probably co-opted and multiplied through several gene duplications during eukaryogenesis; Alg13/Alg14 are more similar to their bacterial homologues; and Alg1, Alg5 and a putative flippase have unknown origins.

**Conclusions:**

The origin of the eukaryotic *N-*glycosylation pathway is not unique and less straightforward than previously thought: some basic components likely have proteoarchaeal origins, but the pathway was extensively developed before the eukaryotic diversification through multiple gene duplications, protein co-options, neofunctionalizations and even possible horizontal gene transfers from bacteria. These results may have important implications for our understanding of the ER evolution and eukaryogenesis.

**Reviewers:**

This article was reviewed by Pr. Patrick Forterre and Dr. Sergei Mekhedov (nominated by Editorial Board member Michael Galperin).

**Electronic supplementary material:**

The online version of this article (doi:10.1186/s13062-016-0137-2) contains supplementary material, which is available to authorized users.

## Background

### At the dawn of eukaryotes

All living organisms are traditionally classified into one of three domains of life, namely bacteria, archaea and eukaryotes. Eukaryotic cells have important cytological specificities, such as nuclei, organelles and other cellular processes. As a result, the origin of eukaryotes from former organisms is one of the most intriguing questions in biology. Endosymbiosis was certainly an important contributor to eukaryogenesis, for at least the mitochondria are known to have evolved from an alpha-proteobacterium that was engulfed prior to the last eukaryotic common ancestor (LECA) [1, 2]. The traditional three-domain tree of life posits that eukaryotes and archaea are sister groups [3–5] and, thus, it assumes that the mitochondrial ancestor was incorporated in a pre-existing proto-eukaryotic lineage [6, 7]. Other phylogenetic studies have suggested that the eukaryotic stem branched within the archaeal domain, close to the crenarchaea–“eocytes”–or to the archaeal TACK superphylum [8–11]. Also, comparative genomics have shown that many eukaryotic operational (metabolic) genes are closely related to bacterial homologues, while informational genes are more similar to archaeal ones [12]. These observations popularized the opinion according to which the eukaryotes are a chimeric lineage that resulted from the endosymbiosis of the bacterial ancestor of mitochondria within a *bona fide* archaeon [13, 14] or a previous bacterium/archaeon consortium [15–17]. The recent accumulation of genomic data has now made possible to address these issues. The debate remains open [18–20], but most authors now favor an archaeal origin of the eukaryotic stem [21–24].

Following the success of comparative analyses to trace back particular machineries to LECA [25–32], the origin and evolution of the eukaryotic *N-*glycosylation pathway will be studied here.

### *N-*glycosylation in the three domains of life

**Fig. 1:**
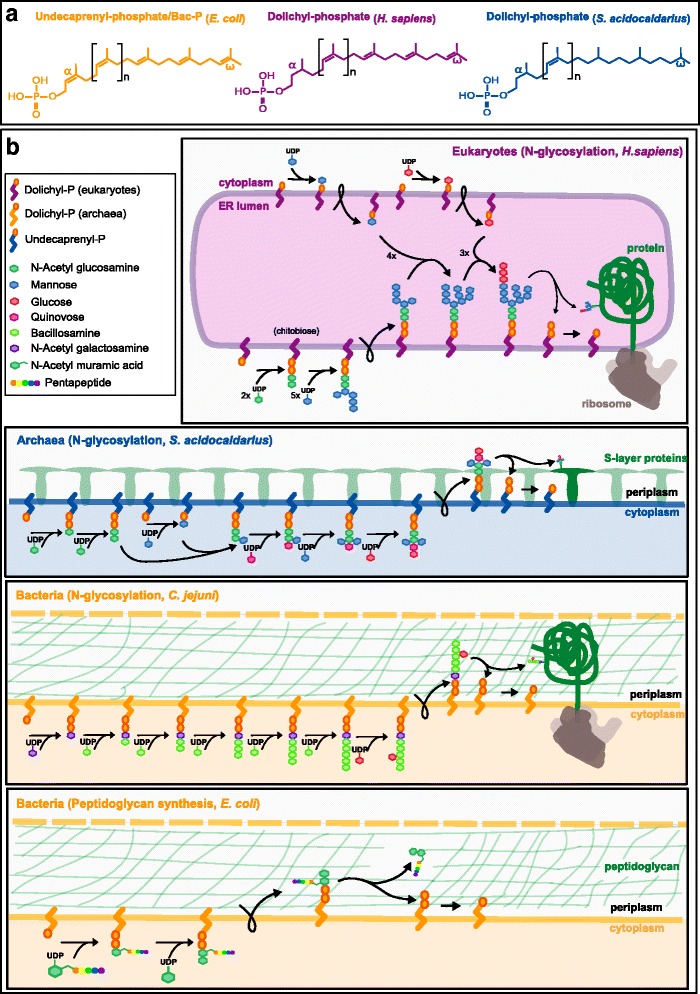
Basic components of the polyisoprenol-based machineries. **a** Typical polyisoprenol lipid carriers in the three domains of life. **b** Topology (membrane orientation) of characteristic *N-*glycosylation pathways in the three domains of life and bacterial peptidoglycan biosynthesis. The lipid carrier is embedded in a cell membrane (ER membrane in eukaryotes, plasma membranes in archaea and bacteria), first facing the cytoplasmic side, then flipped to the opposite side (i.e. ER lumen in eukaryotes, periplasm in prokaryotes). Monosaccharides are attached one by one to the lipid carriers by specific glycosyltransferases, although in the eukaryotic *N-*glycosylation each kind of sugar is only represented once per compartment, for simplicity. The monosaccharides are nucleotide-activated in the cytoplasmic side or translocated to the ER lumen by separate lipid carriers

More than half of all eukaryotic proteins are glycoproteins, and 90 % of those are *N-*glycosylated [33]. Protein *N*-glycosylation modulates protein stability, solubility, rigidity, orientation, interactivity, transport and signaling [34]. It consists of the covalent attachment of an oligosaccharide to the nitrogen atom of specific asparagine residues. In eukaryotes, the synthesis of the oligosaccharide core is mediated by a lipid carrier called dolichol-phosphate (Dol-P) which is located in the membranes of the endoplasmic reticulum (ER, Fig. 1a). In opisthokonts (e.g. in humans and yeast), several cytoplasmic-facing membrane-embedded glycosyltransferases (GTs) sequentially add monosaccharides from soluble nucleotide carriers to Dol-P molecules (Fig. 1b). The Dol-P-oligosaccharide is then “flipped” across the membrane and other membrane-embedded GTs resume the oligosaccharide decoration in the lumen side. The monosaccharides used in the ER lumen are also attached to Dol-P carriers. The final oligosaccharide is transferred *en bloc* to the acceptor protein located in the ER lumen by a *N-*oligosaccharyltransferase (*N-*OST). Soluble GTs located in the ER and Golgi lumen may subsequently modify the protein *N-*glycans [35], but these later modifications will not be studied in the present analysis.

In archaea, flagellins and cell wall S-layer proteins are N-glycosylated using similar pathways located in their cell membranes [36, 37]. Archaeal *N-*glycosylation lipid carriers are traditionally called Dol-P, even though they are more saturated than the eukaryotic ones (Fig. 1a, [38–41]). Archaeal *N-*glycosylation pathways have a topology similar to their eukaryotic counterparts, i.e. the orientation of their reactions across the membrane is the same: archaeal *N-*glycans are synthesized in the cytoplasmic face, then flipped to the outer side of cell membranes and transferred to the protein (Fig. 1b, [37]). Contrary to eukaryotes, however, the *N-*glycan chains are seldom modified in the outer side of archaeal membranes, with the notable exception of *Haloferax volcanii* [42]. Archaeal glycans are also more diverse than their eukaryotic equivalents [40].

*N-*glycosylation is less common in bacteria, where two *N-*glycosylation systems are known [43]. One of them independently tethers single monosaccharides to specific amino acids using soluble cytoplasmic proteins. The other relies on a lipid-carrier-based mechanism similar to the eukaryotic and archaeal pathways (Fig. 1b). The latter is thought to be restricted to delta- and epsilon-proteobacteria [43, 44]. The bacterial polyprenol lipid carriers are unsaturated and have different names depending on their lengths [45]. For simplicity, they will all be referred to as bactoprenol phosphate (Bac-P).

### The evolution of polyisoprenol-based *N-*glycosylations

Since polyisoprenol-based *N-*glycosylation pathways are widespread in archaea and eukaryotes but uncommon in bacteria, it has been speculated that this pathway originated in archaea, was horizontally transferred to some bacteria [46, 47] and inherited in eukaryotes from their putative archaeal-related progenitor [34, 48]. The potential archaeal origin of eukaryotic *N-*glycosylation recently brought this pathway into the spotlight of eukaryogenesis debates [14]. For instance, the “inside-out hypothesis” recently suggested that the eukaryotic nucleus evolved from ancestral “eocytes” (crenarchaeota) that protruded membrane-bound blebs beyond their cell walls to increase the available exchange surface with the bacterial ancestors of mitochondria. According to this hypothesis, the location of the *N-*glycosylation pathway in the eukaryotic ER membranes is a vestige of the ancestral archaeal S-layer protein *N-*glycosylation [14].

The idea that eukaryotic *N-*glycosylation originated from archaea is plausible, but alternative hypotheses have also been suggested. For instance, the eukaryotic *N-*glycosylation has been thought to be related to some kind of bacterial cell wall synthesis pathway [49–51]. Indeed, bacterial *O-*glycosylation, tyrosine *O-*glycosylation, peptidoglycan synthesis, *O*-antigen LPS synthesis, teichoic acid synthesis, exopolysaccharide synthesis and, in a smaller scale, *O*- and C-mannosylation, use polyisoprenol lipid carriers and a similar orientation across the membrane to protein *N-*glycosylation. Other observations, without being conclusive, call into question a simple transposition of the archaeal *N-*glycosylation into eukaryotes: i) archaeal and eukaryotic Dol-P are different molecules (Fig. 1a) [38–41] and they are probably synthesized using different biosynthesis pathways (data to be published soon; Lombard, data not shown); and ii) eukaryotic cytoplasmic GTs have homologues in prokaryotes but the precise evolutionary relationships between eukaryotic and prokaryotic sequences are not known yet [46].

In fact, the available evidence to support a bacterial or archaeal origin of the eukaryotic *N-*glycosylation pathway is limited, so a phylogenomic analysis was carried out to tackle this issue. The phylogenetic study of the involved protein superfamilies suggests that this pathway was present in LECA and that its components have diverse origins. Some genes likely had archaeal ancestors–especially from the TACK superphylum, to which I will refer to as proteoarchaea [52]. Although this gives credit to hypotheses invoking a close relationship between eukaryotes and proteoarchaea, many *N-*glycosylation genes specifically evolved in eukaryotes through gene duplication, protein co-option and neofunctionalization. Two of them could even have bacterial origins. Given the importance that some eukaryogenesis scenarios have conceded to the origins of the *N-*glycosylation pathway and its presence in the ER membranes, these results may have important consequences in debates about the origins of eukaryotes and the ER.

## Results and discussion

### Distribution of the *N-*glycosylation pathway proteins across eukaryotes

The set of *N-*glycosylation GTs was reported to be very variable among eukaryotes [46, 53], therefore raising the question if this pathway was ancestral to all eukaryotes or progressively developed during eukaryotic evolution [46, 48]. The recent accumulation of genomic data allows us to provide a more comprehensive perspective on the distribution of these genes across eukaryotes (Table 1, Additional file 1). With regard to the *N-*OST, the Stt3 catalytic unit is well conserved and most other subunits–except for those very short and arguably more difficult to detect–are found in all major eukaryotic groups but haptophytes and cryptophytes. GTs and putative flippase orthologues are found in all major eukaryotic lineages. The cytoplasmic GTs are more conserved than their lumenal counterparts. This is in agreement with the observation that lumen GTs deletions have milder phenotypes than cytoplasmic GTs mutations [54]. The widespread distribution of the canonical *N-*glycosylation pathway proteins in eukaryotes and their consistent monophyly in protein family trees (see below) support the ancestral presence of this pathway in LECA and the subsequent independent loss of some of these enzymes in some lineages [46]. In parasites, such losses may arguably have been a convenient mechanism to synthesize non-canonical *N-*glycans and escape the host defenses.

**Table 1:** Presence of detectable homologues of the *N-*glycosylation proteins in a diversity of eukaryotes

	Alg7	Alg13	Alg14	Alg1	Alg2	Alg11	Rft1	Alg3	Alg9	Alg12	Alg6	Alg8	Alg10	Stt3	Ost1	Ost2	Ost3/6	Ost4	Ost5	Wbp1	Swp1	Dmp1	Alg5
*Saccharomyces cerevisiae*	+	+	+	+	+	+	+	+	+	+	+	+	+	+	+	+	++	+	+	+	+	+	+
*Schizosaccharomyces pombe*	+	+	+	+	+	+	+	+	+	+	+	+	+	+	+	+	+	+		+	+	+	+
*Ustilago maydis*	+	+	+	+	+	+	+	+	+	+	+	+	+	+	+	+	+			+	+	+	+
*Cryptococcus neoformans*	+	+	+	+	+	+	+	+	+	+				+	+	+	+	+		+	+	+	
*Rhizophagus irregularis*	+	+	+	+	+	+	+	+	+	+	+	+++	+	+	+	+	+			+	+	+	+
*Batrachochytrium dendrobatidis*	++	+	+	+	+	+	+	+	+	+	+	+	+	+	+	+	+			+	+	+	+
*Spizellomyces punctatus*	+	+		+	+	+	+	+	+	++	+	+		+	+	+	+			+	+	+	+
*Allomyces macrogynus*	++	++	++	++	+	++	++	++	+	++	++	++	++	++	++	++	++			++	+	++	++
*Trichoplax adhaerens*	+	+	+	+		+	++		+	+	++	+	+	++	+	+	+			+	+	+	+
*Amphimedon queenslandica*	+	+++	+	+	+	+	+	++	+	+	+	+++	+	5+	+	+	+++	+		+	+	+	+
*Mnemiopsis leidyi* ^a^	+	+	+	+	+	+			+	+		+	+	++	+	+	+			+	+		+
*Hydra magnipapillata*	+	+	+	+	+	+			+	+		+	+	++	+	+	+			+	+		+
*Hydra magnipapillata*	+	+	+	+	+	+	+	+	+	+	+	+	+	+++	+	+	++			+	+	+	+
*Drosophila melagonaster*	+	+	+	+	+	+	+	+	+	+	+	+	+	++	+	+	+	++		+	+	+	+
*Aplysia californica*	+	+	+	+	+	+	+	+	+	+	+	+	+	+++	+	+	+	+		+	+	+	++
*Strongylocentrotus purpuratus*	+	+	+	++	+	+	++	+	+	+	+	+	+	+++	+	+	++			+	+	+	+++
*Mus musculus*	+	++	+	++	+	++	+	+	+	++	+	+	+	++	+	+	++	+		+	++	+	+
*Monosiga brevicollis*	+	+	+	+	+	+	+	+	+	+				++								+	
*Salpingoeca rosetta*	+	+	+	++	+	+	+	+	+	+	+	+	+	++	+	+	+			+	+	+	+
*Capsaspora owczarzaki*	+	+	+	+	+	+	+	+	+	+	+	++	+	++	+	+	+	+		+	+	++	+
*Fonticula alba*	+	+	+	+	+	+	+				+	+		+	+	+	+			+		+	+
*Thecamonas trahens*	+	+	+	+	+	+	+	+	+	+	+	+	+	++	+	+	+			+	+	+	+
*Entamoeba histolytica*	+	+	+	+	++	+	+							+++	++	+				+++		+	
*Acanthamoeba castellanii*	+	+	+	+	+	+	+	+	+		+	+	+	+		+		+			+	+	+
*Dictyostelium discoideum*	+	+	+	+	+	+	+	++	+	+	+	+	+	+	+	+	+	+		+	+	+	++
*Polysphondylium pallidum*	+	+	+	+	+	+	+	++	+	+	+	+	+	+	+	+	++	+		+	+	+	+
*Chlamydomonas reinhardtii*	+	+	+	+	+	+	+				+	+		++	+	+	+			+	+	+	++
*Volvox carteri*	+	+	+	+	+	+	+				+	+	+	++	+	+	+			+	+	+	+
*Chlorella variabilis*	+	+	+	+	+	+	+		+	+	+	+	+	+	+	+	+			+	+	+	+
*Asterochloris* sp.	+	+	+	+	+	+		+	+	+	+	+		+	+	+	+			+		+	+
*Bathycoccus prasinos*	+	+	+	+	+	+	+	+	+	+				+								+	
*Micromonas pusilla*	+	+	+	+	+	+		+	+	+	+	+		++								+	+
*Ostreococcus lucimarinus*	+	+	+	+	+	+	+	+	+	+				+								+	+
*Klebsormidium flaccidium*	+	+	+	+	+	+	+	+	+	+	+	+	+	++	+	+	+			+	+	+	+
*Physcomitrella patens*	+	+	+	+	+	++	++	+	+	++	+	+	++	+++	+	++	+++	+		+	+	++	+++
*Selaginella moellendorffii*	4+	+	++	+	+	+	++	+	+	+	+	+	+	++	++	+	4+			++	++	+	4+
*Pinus taeda* ^a^		+				+++			+		+	+	+	5+	+	++		+		+	+	+	
*Amborella trichopoda*	+	+	+	+	+	+	+	+	+	+	+	+	+	++	++	+	+	+		+	+	+	+
*Oryza sativa*	+	+	+	++	+	+	+	+	+	+	+	+	+	++	++	+	+	+		+	+	+	+
*Aquilegia coerulea*	+++	++	+	+++	++	+++	++	+	+	++	++	+	+	++	+++	++	+	++		+++	+++	++	++
*Prunus persica*	++	++	+	++	++	+	++	+	+		+	++	+	++	++	+++	+	+		++	+	+	+
*Arabidopsis thaliana*	++	+	+	++	+	+	+	+	+	++	+	+	+	++	++	+++	++	++		+	++	+	++
*Calliarthron tuberculosum* ^a^	+			++	+		+		+	+	+	+	+	+	+					+		+	
*Chondrus crispus*	++	+		+	+	+	+	+		+	+	+	+	+		+	+			+		+	
*Cyanidioschyzon merolae*	+	+	+	+	+	+	+	+	+	+	+	+	+	+	+	+				+		+	+
*Galdieria sulphuraria*	+	+		+	+	+	+	+	+	+	+	++	+	+	+	+				+	+	+	+
*Porphyridium purpureum*			+	+	+	+	+	+	++	+	+	+	+	+++	+	+				+	+	+	+
*Pyropia yezoensis* ^a^	+							+	+					+	+	+				+		+	
*Cyanophora paradoxa* ^a^	+	+		+	+	+		++	++		+	+	+	+		+	+			+	+	++	+
*Phaeodactylum tricornutum*	+	+	+	+	+	+	+	+	+	+	+	+		++								+	4+
*Blastocystis hominis*	+	+	+	+	+	++	+	+	+	+	+	+	+	+	+	+	+			+	+	+	+
*Phytophthora parasitica*	+	+	+	+	+	+	+	+	+	+	+	++	++	++	+	+	++			+	+	+	+
*Schizochytrium aggregatum*	+	+	+	+	+	+	+	+	+	+	+	+		+								++	+
*Aureococcus anophagefferens*	+	+	+	+	+	+		+			+	+		++								+	+
*Plasmodium falciparum*	+	+	+											+	+					+		+	
*Babesia equi*	+	+	+	+										+	+							+	
*Theileria annulata*	+																					+	+
*Toxoplasma gondii*	+	+	+	+	+						+	+	+	+	+	+	+			+		+	++
*Gregarina niphandrodes*	+	+		+	+	+								+								+	
*Ichthyophthirius multifiliis*			+	+	+	+	+				+	+		+	+		+			+		+	+
*Tetrahymena thermophila*	+	+	+	+	+	+	+				+	+	+	+	+	+	+			+	+	+	+
*Oxytricha trifallax*	+	+	++	+	+	+	+	+		+	+	+		+	+	+	+			+		+	+
*Paramecium tetraurelia*	+	+	++	+	+	+	+				+	+	+	+	++	++	++			++	+	++	++
*Symbiodinium minutum*		+	+	+	+	+		+	+		+	++	+	6+								+	+
*Perkinsus marinus*	++			+	++	+		++	++	++	+	++	+	++	++	+	++			++	+	+++	++
*Bigelowiella natans*	+	+	+	+	+	+	+	+	+	+	+		+	+	+	+	+			+	+	+	+
*Reticulomyxa filosa*	+	+		+	+	+	+	+	+	+	+	++	++	+	+	+	+				+	+	+
*Bodo saltans*	+	+	+	+	+	+	+	+	+	+	+	+	+	4+								+	+
*Angomonas deanei*	+	++	++		+		+	++						5+								+	
*Leishmania major*	+	+	+	+	+	+	+	+	+					4+								+	+
*Trypanosoma vivax*	+	+	+	+	+	+	+	+	+	+				+++								+	
*Giardia lamblia*	+	+	+											+								+	
*Spironucleus salmonicida*	+	+	+											+									
*Naegleria gruberi*	+	+	+	+	+	+	+	+	+	+	+	+	+	+	+	+	+			+	+	+	+
*Trichomonas vaginalis*	+	+	+	+++	+	+	+							5+	+	+				+		+	5+
*Emiliania huxleyi*	+		+		+	++	+	++	+	+	+	+		4+								+	5+
*Guillardia theta*	+	+	+	+	+	+	+	+	+	+	+	+	+	+++								+	+

^a^Failure in homologue detection may result from genome incomplete sequencing. (+) Presence of homologues in a genome (two or more (+) indicate as many paralogues). (F) on Alg14/Alg13 point out organisms in which the two genes are fused

### Gene homology across polyisoprenol-based machineries

The main argument put forward to suggest bacterial or archaeal origins to the eukaryotic *N-*glycosylation pathway has been the use of polyisoprenol carriers and topological similarity, i.e. the eukaryotic pathway has the same functional orientation across the membrane than several prokaryotic glycosylation pathways (Fig. 1b). Despite these similarities, the homology of the proteins implicated in these pathways had never been systematically surveyed before.

Most proteins involved in polyisoprenol-based pathways are glycosyltransferases (GTs). These enzymes are very common and they are classified according to their structure, catalytic mechanism and amino acid sequences [55–58]. An updated classification is available in the CAZy database (http://www.cazy.org/GlycosylTransferases.html) which, as of August 2016, contained 100 GT families. Nonetheless, their extreme diversity, poor sequence conservation and ability to easily change their substrate specificity make an extensive in-depth evolutionary study of all GTs across the three domains of life virtually impossible.

The pathways summarized in Table 2 were selected for this study because, according to the literature, they use polyisoprenol lipid carriers and have similar topologies to the eukaryotic *N-*glycosylation pathway. The eukaryotic GPI pathway was included notwithstanding being polyisoprenol-independent because its mannosyltransferases are the only known relatives of the eukaryotic *N-*glycosylation lumen GTs [59].

**Table 2:** Polyisoprenol-related pathways used in this analysis and respective literature

*N-*glycosylation	Eukaryotes (*S. cerevisiae*)	KEGG: http://www.genome.jp/kegg/
GPI synthesis	Eukaryotes (*S. cerevisiae*)	KEGG: http://www.genome.jp/kegg/
*O-*mannosylation	Eukaryotes (*H. sapiens*)	KEGG: http://www.genome.jp/kegg/ [87]
*N-*glycosylation	Archaea (*S. acidocaldarius*)	[45]
*N-*glycosylation	Archaea (*M. maripaludis*)	[36, 40]
*N-*glycosylation	Archaea (*M. voltae*)	[36, 71]
*N-*glycosylation	Archaea (*A. fulgidus*)	[50]
*N-*glycosylation	Archaea (*H. volcanii*)	[36, 40]
*N-*glycosylation	Archaea (*H. volcanii,* alternative)	[113]
*N-*glycosylation	Bacteria (*C. jejuni*)	[43]
Peptidoglycan synthesis	Bacteria (*E. coli*)	[114]
LPS synthesis (ABC transporter)	Bacteria (*E.coli*)	[115]
LPS synthesis (wzy-dependent)	Bacteria (*S. enterica*)	[115]
Wall teichoic acids	Bacteria (*B. subtilis*)	[116]
*O-*glycosylation	Bacteria (*N. gonorrhoae*)	[117]
*O-*glycosylation	Bacteria (*G. stearothermophilus*)	[118]
Tyrosine *O-*glycosylation	Bacteria (*P. alvei*)	[101]
Enterobacterial common antigen	Bacteria (*E. coli*)	[119]
Capsule synthesis	Bacteria (*E. coli*)	[120]
EPS - xanthan	Bacteria (*X. campestris*)	[121]
EPS - succinoglycan	Bacteria (*R. meliloti*)	[122]
EPS	Bacteria (*L. lactis*)	[73]

**Fig. 2:**
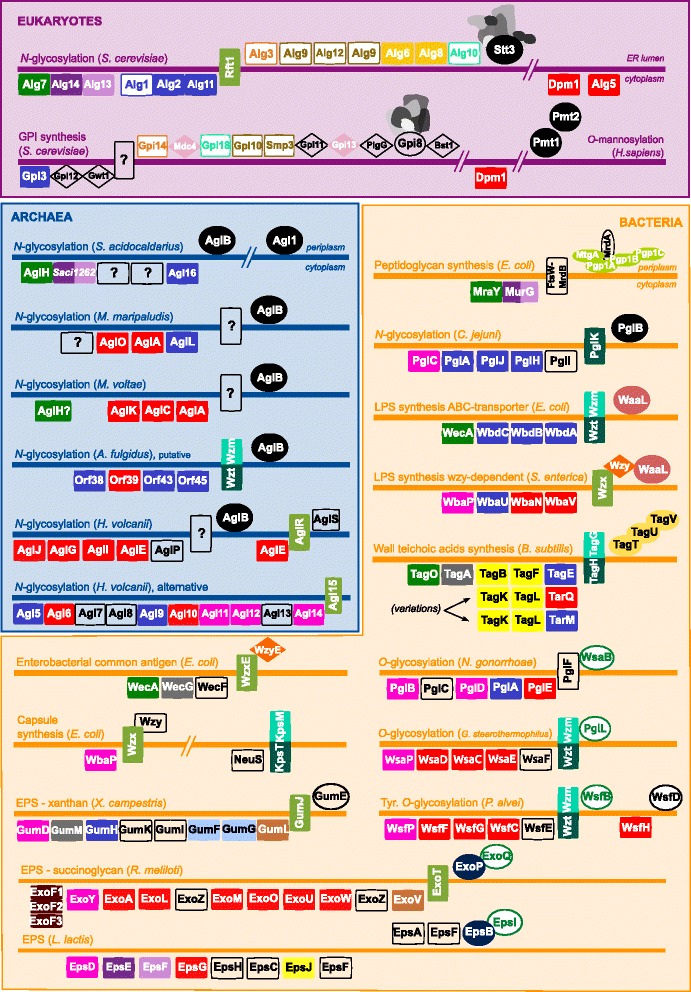
Polyprenol-based glycosylation pathways (and GPI biosynthesis) colored according to detected homology groups. Horizontal lines represent ER membranes in the eukaryotes, plasma membranes in prokaryotes. Horizontal rectangles represent cytoplasmic glycosyltransferases (GTs) if they are below the membrane or lumen/periplasmic GTs if they are above the membrane. Vertical rectangles depict flippases or translocation mechanisms. Ovals represent the oligosaccharide transferases from the lipid carrier to the acceptor molecule. Diamonds portray proteins that are neither GTs nor translocases (e.g. acetyl or ethanolamine transferases in GPI biosynthesis). Extra shapes in the eukaryotic oligosaccharide transferases reflect the fact that these are complexes with many subunits. The cytoplasmic GTs depicted after a transfer to the acceptor molecule (e.g. Dpm1 and Alg5 in eukaryotic *N-*glycosylation) represent polyisoprenol-P-monosaccharide synthases tranfering single mannoses or glucoses to a lipid carrier to supply these sugars to the opposite side of the membrane. Proteins are colored according to the homology group to which they belong (as defined in Methods). Plain symbols represent proteins that were detected using the procedure described in Methods, whilst empty white shapes show more distant relationships that required bibliographic or extra analyses to be established. Empty transparent shapes represent the lack of detection of any homologues in the dataset

The proteins in the pathways from Table 2 were used to carry out all vs. all reciprocal BLASTp searches [60]. The results, together with some information from the literature and occasional HMMER confirmations, allowed the construction of the putative homology groups reported in Fig. 2 (see Methods). This dataset is limited to the pathways characterized in model organisms and does not have a strong statistical value. Yet, it shows that most proteins from polyisoprenol-dependent pathways belong to a limited number of homology groups. For instance, the GTs colored in blue (GT group 1), red (GT group 2) and green (HPT family), plus the khaki flippase group, are present in pathways from the three domains of life, including the eukaryotic *N-*glycosylation pathway. Some pathways from closely related organisms contain the same homology groups. This is for example the case of the *O-*glycosylation in *G. stearothermophilus* and Tyr. *O-*glycosylation in *P. alvei* (two Firmicutes)*; N-*glycosylation in *H. volcanii* and *M. voltae* (two Euryarchaeota)*;* or *O-*antigen ABC-dependent synthesis in *E. coli* and *N-*glycosylation in *C. jejuni* (two Proteobacteria). It is more difficult to ascertain clear parallels across pathways from distantly-related organisms. Nevertheless, some proteins of the eukaryotic *N-*glycosylation pathway belong to the same homology groups than proteins implicated in the peptidoglycan synthesis, LPS synthesis or putative *N-*glycosylation in *A. fulgidus*. The most exciting parallel is to be drawn between the eukaryotic and putative *S. acidocaldarius N-*glycosylation pathways. This Sulfolobus pathway has not been fully described yet [45], but it is the only prokaryotic mechanism studied here with homologues of all eukaryotic cytoplasmic GTs and a catalytic *N-*OST subunit. This result is suggestive because many eukaryogenesis scenarios assume a tight evolutionary relationship between proteoarchaea and eukaryotes [8–10, 14] but it must be balanced with the phylogenetic analyses presented below.

### The eukaryotic *N-*glycosylation initiation complex has a composite origin

The canonical eukaryotic *N-*glycosylation pathway starts with the sequential link of two N*-*acetylglucosamines (GlcNAc) from soluble UDP-GlcNAc to Dol-P (Fig. 1, [61, 62]). The resulting GlcNAc dimer is named chitobiose. The two GlcNAc transfers are consecutively carried out by Alg7 and Alg13, which form a protein complex with the membrane anchor Alg14 [51, 63, 64]. Alg7 belongs to the polyprenol phosphate:N-acetylhexosamine-1-phosphate transferase (HPT) family (dark green in Fig. 2, [65–67], which also includes the bacterial MraY protein. Alg14 and Alg13 are respectively homologous to the N- and C-terminal parts of bacterial MurG (purple in Fig. 2, [62]). MraY, MurG and other proteins form a complex involved in peptidoglycan synthesis (Fig. 2, [51, 68]). The resemblances between the eukaryotic Alg7-Alg13-Alg14 and the bacterial MraY-MurG complexes [69] could support the existence of a close evolutionary relationship between the eukaryotic *N-*glycosylation and the bacterial peptidoglycan synthesis pathways [49, 51]. Although most archaeal *N-*glycosylation initiation mechanisms are based on GTs from group 2 (red GTs in Fig. 2, [36]), HPT homologues have been reported in several archaea [45, 70]. For instance, the *S. acidocaldarius* genome contains both a HPT and a *murG* homologue, and the *N-*glycans in this organism are also initiated with a chitobiose [45]. Despite these promising similarities between the eukaryotic and the putative *S. acidocaldarius N-*glycosylation pathways (Fig. 2, [45]), the actual role of the archaeal HPT proteins is debated [70, 71] and the MurG homologue remains uncharacterized to this date.

The phylogeny of each protein involved in the initiation complex was carried out. That of the HPT superfamily shows two large groups (Bayesian Posterior Probability, BPP = 1), one mostly bacterial and the other including most archaeal and eukaryotic sequences (Additional file 2). The phylogeny of the bacterial clade is discussed in detail the Additional file 2 but, in summary, it supports the idea that at least two proteins from the HPT superfamily were present in the Last Bacterial Common Ancestor (LBCA). Here, we will center our attention on the other cluster, for it contains the eukaryotic sequences.

**Fig. 3:**
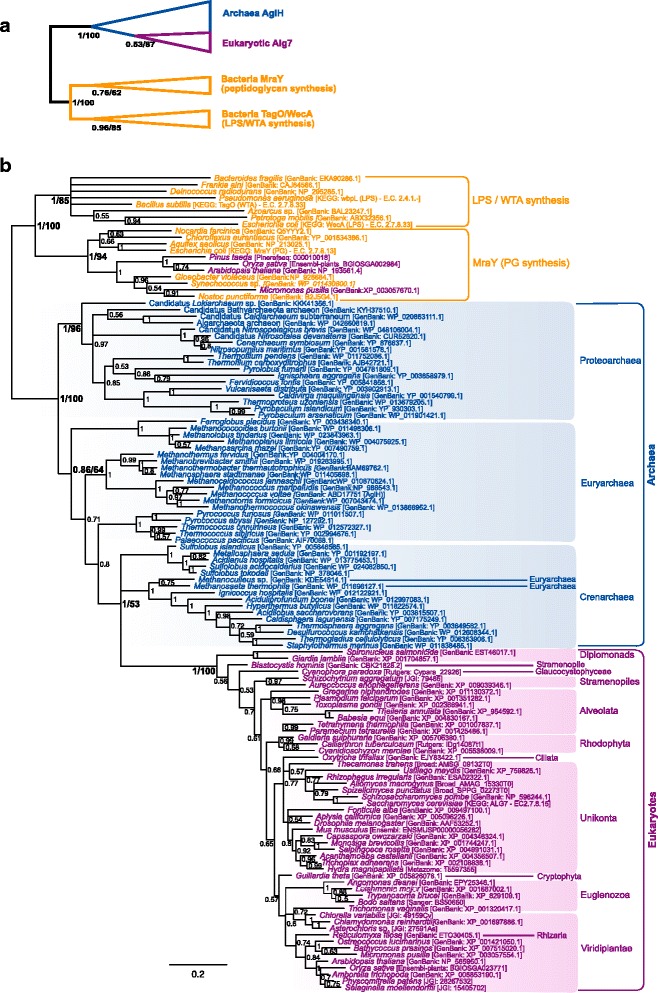
Bayesian phylogeny of the HPT homologues. **a** Schematic phylogenetic tree including the bacterial sequences (see Additional file 2 for details). **b** Specific phylogeny of the archaeal/eukaryotic clade. The tree was reconstructed using 124 representative sequences and 203 conserved sites. Multifurcations correspond to branches with Bayesian posterior probabilities <0.5. Numbers at nodes indicate Bayesian posterior probabilities higher than 0.5. Bootstrap values from maximum likelihood analyses are reported on basal and major nodes. Colors on leaves represent the affiliation of sequences to a domain of life: archaea (*blue*), bacteria (*orange*) and eukaryotes (*purple*)

A specific phylogenetic analysis only focusing on this part of the tree was carried out (Fig. 3). It shows a monophyletic group of eukaryotes (BPP = 1) in which sequences cluster according to meaningful taxonomic groups, such as paraphyletic groups of unikonta, viridiplantae or alveolates. A eukaryotic-only phylogeny revealed a better resolution and an overall topology in accordance with the main eukaryotic taxa (e.g. monophyly of unikonts, archaeplastida, excavates, etc. Data not shown). This suggests that the lack of resolution among eukaryotes in the Fig. 3 may be due to reconstruction artifacts owing to the extreme divergence between the prokaryotic and the eukaryotic sequences. The wide distribution of these genes in eukaryotes and the phylogenetic clustering of the eukaryotic sequences according to their taxonomic groups support the presence of Alg7 in LECA. Moreover, the eukaryotic clade branches within a paraphyletic group of crenarchaea (BPP = 1) which itself branches in a paraphyletic group of euryarchaea (BPP = 0.86). A proteoarchaeal clade forms a sister group to the rest of archaeal and eukaryotic sequences (Fig. 3). The existence of the basal proteoarchaeal gene may suggest that the Last Archaeal Common Ancestor (LACA) had two paralogues of this family, one of which was lost in the euryarchaeal lineage. Alternatively, the rampant paraphyly of this tree may be indicative of some reconstruction artifacts due to the lack of phylogenetic signal or inappropriate phylogenetic reconstruction models. In that case, the crenarchaeal gene could result from a phylum-specific duplication, and the basal branching of the proteoarchaeal group could result from a long branch attraction artifact. Regardless of the origin of the two proteoarchaeal paralogues, at least one representative of the HPT family seems to have been present in both the last common ancestor of euryarchaea and proteoarchaea and, thus, possibly in LACA. The eukaryotic Alg7 orthologues unambiguously branch within the paraphyletic crenarchaeal group–a group that also includes *S. acidocaldarius* putative AglH (HPT) protein. Yet, there are no obvious closest crenarchaeal relatives to the eukaryotic clade. Thus, although the eukaryotic Alg7 genes likely have a proteoarchaeal origin, the precise identity of this ancestor remains uncertain.

**Fig. 4:**
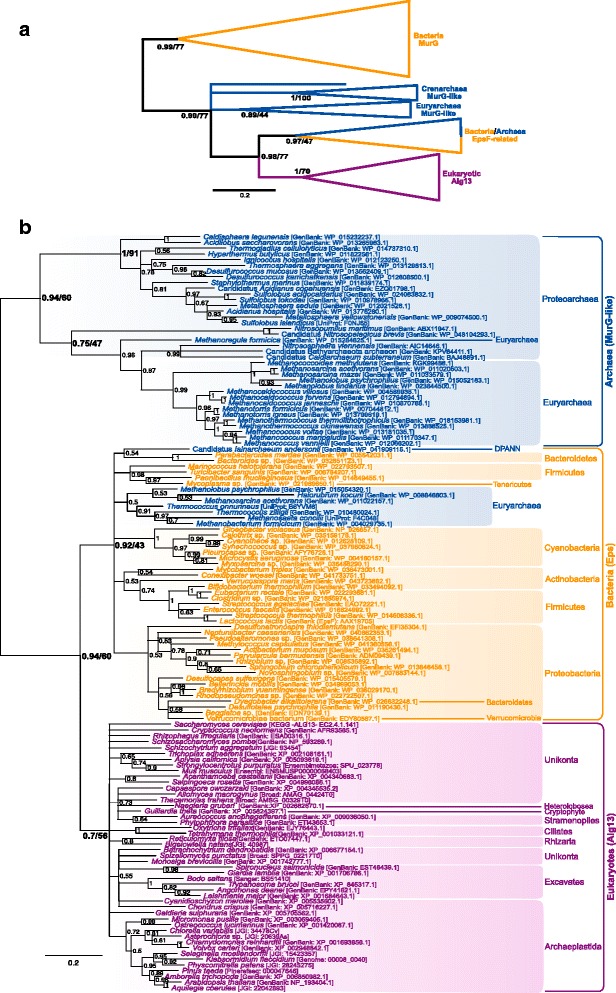
Bayesian phylogeny of the Alg13/catalytic MurG domain. **a** Schematic phylogenetic tree including the bacterial MurG homologues (see Additional file 3 for details). **b** Specific phylogeny excluding the bacterial MurG clade. The tree is unrooted and was reconstructed using 132 representative sequences and 116 conserved sites. Multifurcations correspond to branches with Bayesian posterior probabilities <0.5. Numbers at nodes indicate Bayesian posterior probabilities higher than 0.5. Bootstrap values from maximum likelihood analyses are reported on basal and major nodes. Colors on leaves represent the affiliation of sequences to a domain of life: archaea (*blue*), bacteria (*orange*) and eukaryotes (*purple*)

Regarding the MurG/Alg14/Alg13 homologues, several occurrences of split or fused genes exist. For instance, the heteromer EpsE/EpsF involved in the exopolysaccharide (EPS) synthesis of firmicutes [72, 73] has been reported to be split into the same protein domains as Alg14/Alg13 [62]; in the contrary, excavates and some amoebozoa have fused Alg14-Alg13 polypeptides (Table 1). In order to account for these different architectures, independent phylogenies were constructed for each protein domain: catalytic (Alg13/C-terminal part of MurG) on the one side and membrane anchor (Alg14/N-terminal part of MurG) on the other. In both phylogenies, MurG homologues were widespread among bacteria and formed a highly divergent clade (BPP = 0.99 in both cases, Fig. 4a, Additional files 3 and 4). The bacterial MurG phylogeny is discussed in more detail in the Additional files 3 and 4 but, in short, MurG was likely already present in the LBCA.

Phylogenies excluding the bacterial MurG homologues were carried out to more precisely tackle the evolution of the closest prokaryotic relatives to the eukaryotes (Fig. 4b and Additional file 5). This includes archaeal fused sequences (referred to as MurG-like) and bacterial and archaeal split (EPS-like) sequences. The independent phylogenies of both the catalytic and membrane anchor domains show similar results to each other: three clades corresponding to 1) the archaeal fused MurG-like homologues (BPP Alg13 = 0.94, BPP Alg14 = 0.67); 2) the bacterial and archaeal split EPS-like homologues (BPP Alg13 = 0.92); and 3) the eukaryotic proteins (BPP Alg13 = 0.7, BPP Alg14 = 0.87). The fused MurG-like archaeal clade is divided into a crenarchaeal group and a euryarchaeal group with some basal thaumarchaeota and bathyarchaeota. Despite the paraphyly of the proteoarchaeal sequences (i.e. the crenarchaeal and thaumarchaeal sequences in this tree), the wide distribution of these proteins in archaea and the monophyly of the crenarchaeal and euryarchaeal sequences supports the presence of this gene in LACA. The internal topology of the eukaryotic clade is very mixed and very poorly resolved even when phylogenies are reconstructed only with eukaryotic sequences (data not shown). This could translate a complicated non-vertical evolutionary history for these genes but it most likely results from the small number of alignment positions that was conserved in each phylogeny (116 and 97 respectively) and the fast evolution rate of these proteins, especially the membrane anchor. Despite these difficulties, the wide distribution of Alg13/Alg14 orthologues across eukaryotes and their monophyly in all the phylogenies suggest that these proteins may have been ancestral to eukaryotes. Finally, the split EPS-like prokaryotic sequences are dominated by bacteria. The archaeal split EPS-like sequences are few and scattered among the bacterial sequences, so they certainly result from HGTs. The EPS-like bacterial sequences form meaningful taxonomic groups, but their distribution across bacteria is not very diverse. Thus, unlike MurG, the ancestral presence of EPS-like genes in the LBCA is uncertain.

The relationship of the eukaryotic clades to their prokaryotic relatives is more difficult to establish. The eukaryotic sequences form separate clades with regard to the other domains of life, so these phylogenies could be discussed in terms of a three-domain of life topology. This line of thought would suggest that the eukaryotic Alg13/Alg14 proteins were inherited from the last common ancestor of extant organisms (i.e. the cenancestor or LUCA) through a proto-eukaryotic lineage. Nevertheless, if LBCA and LACA had fused MurG proteins, as this analysis supports, it follows that whatever the topology of the tree of life, the cenancestor most likely had a fused MurG-like protein. This is not the case of the split proteins, of which the distribution among prokaryotes is too scarce to support their presence in the cenancestor. Since, contrary to bacteria, the eukaryotes do not have other homologues of the Alg13/Alg14 proteins, the most parsimonious hypothesis for the origin of the split genes would be that *epsE/epsF* developed in the bacterial lineage from the duplication and subsequent split of their ancestral *murG* gene and were later acquired in an early eukaryotic ancestor from an unidentified bacterium. Once in the eukaryotic lineage, they evolved into the *alg13/alg14* couple.

In summary, no evidence was found to support a direct relationship between the bacterial peptidoglycan synthesis and the eukaryotic *N-*glycosylation pathway. The eukaryotic HPT was present in LECA and likely inherited from proteoarchaea. The Alg13/Alg14 couple is structurally more similar to their bacterial homologues and, thus, could have a bacterial origin. These results highlight the mixed origin of the eukaryotic *N-*glycosylation initiation complex. Furthermore, since at least one representative of both the HPT and MurG families was present in the respective common ancestors of both prokaryotic domains, their presence may also be inferred in the cenancestor. This suggests that the cenancestral membranes may have had the basic elements of a glycosylation machinery. Given the fact that most modern prokaryotic polyisoprenol-based glycosylation pathways are involved in the synthesis of prokaryotic cell wall components, this may suggest that the cenancestor was already bound by some kind of glycosylated envelope, of which the specific nature is unknown. The implications of these results for the possible cenancestral cell walls will be discussed in a paper to be published soon (Lombard, data not shown).

### Cytoplasmic mannosylation evolved from an archaeal protein

After the chitobiose core, five mannoses are tethered to the growing glycan in the cytoplasmic face of the ER membrane (Fig. 1b). The first mannosylation is generally ascribed to Alg1, the second and third to Alg2 and the two last to Alg11, but their activity depends on the formation of respective protein complexes: Alg1 homooligomers, respective Alg2-Alg1 and Alg11-Alg1 heteromers [54]. All three proteins belong to the glycosyltransferase 1 superfamily (GT1), even though Alg1 is classified as a separate family in the CaZY database [54]. These eukaryotic genes were suggested to have archaeal ancestors [50], but early phylogenetic analyses were unable to support this hypothesis. Thus, these proteins had also been postulated to be eukaryotic-specific [46].

The GT1 superfamily is widely represented in most polyisoprenol-based pathways in the three domains of life (dark blue proteins in Fig. 2). The high number of homologues of the GT1 enzymes detected in the three domains of life proved challenging to handle, so preliminary analyses were carried out to exclude the most distant GT1 homologues from the analysis and concentrate in the closest relatives to the eukaryotic sequences (see Methods). These preparatory analyses showed that the Alg1 sequences formed a monophyletic group but their branching position within the superfamily tree was highly inconsistent and depended on the sequences selected for the analysis (data not shown). As a result, the closest prokaryotic relatives of the eukaryotic *alg1* could not be determined. The origin of this gene could not be assessed, and this may suggest that Alg1 was a eukaryotic innovation. A tentative explanation of the high Alg1 divergence–even when compared to the outstanding diversity of GT1 members–could be the role of Alg1 in the formation of the mannosyltransferase complexes [54].

**Fig. 5:**
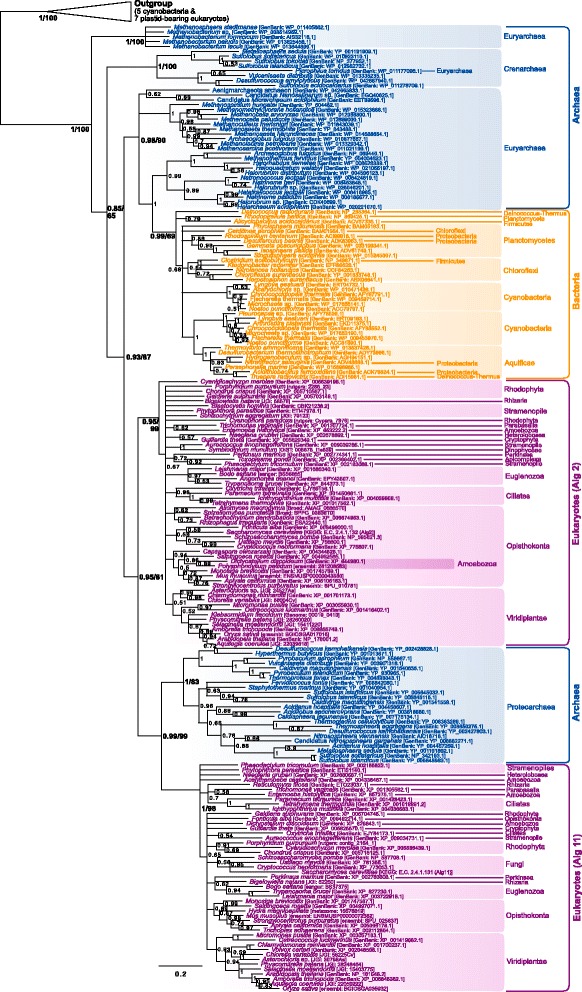
Bayesian phylogeny of the closest relatives to the eukaryotic Alg2/Alg11 homologues. The tree is unrooted and was reconstructed using 211 representative sequences and 194 conserved sites. Multifurcations correspond to branches with Bayesian posterior probabilities <0.5. Numbers at nodes indicate Bayesian posterior probabilities higher than 0.5. Bootstrap values from maximum likelihood analyses are reported on basal and major nodes. Colors on leaves represent the affiliation of sequences to a domain of life: archaea (*blue*), bacteria (*orange*) and eukaryotes (*purple*)

Contrary to Alg1, Alg2 and Alg11 are closely related to each other and to some prokaryotic sequences. Their evolution was studied together with their closest prokaryotic relatives (see Methods). The outgroup of this analysis is made up of some sequences from plastid-bearing eukaryotes and cyanobacteria (Fig. 5). The first sequences to diverge from the outgroup are some Methanobacteriales and a group dominated by crenarchaea. The position of these sequences frequently changed in the preliminary trees (data not shown) and their basal position in the Alg2/Alg11 tree calls into question their closeness to the proteins that interest us. Nevertheless, the lack of characterization of these genes makes it difficult to make an informed decision to exclude them from the analysis. The rest of the tree is split in four clades: a euryarchaeal clade also including several bacterial groups (BPP = 0.98), a proteoarchaeal clade (BPP = 1) and two eukaryotic clades respectively corresponding to Alg2 (BPP = 0.95) and Alg11 (BPP = 1). The deep relationships within the eukaryotic clades are unresolved, but their monophyly and widespread distribution suggests that both genes were inherited from LECA. The Alg2 sequences form a sister group to a clade made up of the eukaryotic Alg11 and most proteoarchaeal sequences. Several scenarios may explain this topology. One possibility is that an ancestor of the proteoarchaea horizontally acquired one of these genes from eukaryotes early in proteoarchaeal evolution. Yet, since these homologues are present in a wide diversity of archaea and form respective monophyletic groups of euryarchaea and proteoarchaea, it seems likely that at least one of these proteins was present in LACA. In that case, interpretations depend on the topology of the tree of life which is favored. In the context of a traditional tree of life, in which archaea and eukaryotes are sister groups, the topology could be explained by a hidden paralogy: the last common ancestor of both domains had two paralogues, the eukaryotic linage kept both of them and the proteoarchaea and euryarchaea alternatively lost one of them. Nevertheless, the requirement of Alg1 to form functional heteromers with Alg2/Alg11 contradicts the ancestral paralogy of these proteins in archaea, as Alg1 is a eukaryotic innovation. Alternatively, the ancestor of the eukaryotic (and bacterial) sequences that branch within the archaeal clade could have been acquired from archaea. Since Alg11 is more closely related to the proteoarchaeal sequences, it seems more likely that the original eukaryotic GT1 gene had proteoarchaeal origins, a hypothesis that would also be congruent with the currently favored symbiogenic origin of eukaryotes [21–24]. In that case, the original eukaryotic GT1 gene would have been duplicated and acquired their *alg2/alg11* functions in eukaryotes. The fact that both eukaryotic clades do not form relative sister groups within the proteoarchaea could be a reconstruction artifact due to the mutational saturation that resulted from the development of heteromers and new functions in eukaryotes.

The characterization of the closest archaeal relatives to the eukaryotic Alg2/Alg11 proteins will be instrumental to the evaluation of these different evolutionary hypotheses. ORF43 has been suggested to be involved in *N-*glycosylation in *A. fulgidus* but, in fact, the closest archaeal relatives to Alg2/Alg11 are uncharacterized GT1 proteins [50]. Agl16, a GT1 protein implicated in *N-*glycosylation in *S. acidocaldarius* [74] is not present among the closest relatives of Alg2/Alg11. Similarly, all the GT1 homologues from the Lokiarchaeum metagenome that has been suggested as the closest archaeal relative to eukaryotes [11] belong to other parts of the general (preliminary) GT1 superfamily tree. Until these archaeal proteins are characterized, the lack of functionally important Alg1 in archaea and currently favored symbiogenic scenarios for the origin of life rather favor the proteoarchaeal origin of the eukaryotic *alg2/alg11* GT1 ancestor.

In summary, Alg2 and Alg11 could have evolved from a proteoarchaeal GT1 ancestor and Alg1 has unknown origins. Modern eukaryotic complexes based on Alg1, Alg2 and Alg11 are eukaryotic innovations developed prior to LECA.

### The unclear origin of an uncertain flippase

The transport of polyisoprenol-linked glycans across the membrane is generally thought to be mediated by proteins called flippases [75]. Rft1 was suggested to be the eukaryotic *N-*glycosylation flippase [76] but the actual activity of this protein has been called into question [77–79]. Rft1 has homologues in the three domains of life (Fig. 2). The phylogeny of the Rft1 homologues shows that the eukaryotic sequences form a monophyletic group (BPP = 0.99) supportive of their inheritance from LECA, but it is unable to clarify the relationship of the eukaryotic proteins with their prokaryotic relatives (Additional file 6). Thus, the origin of the eukaryotic Rft1 is unclear (see Additional file 6 for a more detailed discussion).

### The lumen GTs are related to the protein *O-*mannosyl transferases (PMTs)

Once translocated to the lumen side of the ER membrane, the Dol-P-linked glycan is mannosylated four times and glucosylated three times to make up the canonical opisthokont core glycan (Fig. 1b). These modifications are catalyzed by the mannosyltransferases Alg3, Alg9 and Alg12 and the glucosyltransferases Alg6, Alg8 and Alg10. These proteins appear to be unique to eukaryotes [46, 50], so their evolution has only been studied in this domain [59]. The closest relatives of these GTs are the eukaryotic mannosyltransferases involved in the GPI biosynthesis (Fig. 2, [59]), which also takes place in the lumen side of the ER membrane [80]. All the lumen GTs have similar multispan transmembrane domains that have been compared to the topology of sugar transporters [59]. Lumen GTs were classified into three superfamilies according to the sugar they used, the sugar linkage they catalyze and their common peptide motifs. Gene duplication and neofunctionalization events would explain the emergence of the plethora of specialized GTs known today [59].

In the current analysis, the study of the lumen GTs was extended to a much larger diversity of eukaryotes and found to be widespread in most eukaryotic lineages (Table 1, Additional file 1). The phylogenies of each superfamily support the monophyly of each lumen GT and, thus, its likely inheritance from LECA (data not shown). This analysis also suggests a previously unreported possible relationship between Alg10 and Gpi18 (data not shown). Nevertheless, the sequences from different superfamilies are so divergent that alignments comprising several superfamilies are highly unreliable (Guidance alignment score < 0.1), and so are the resulting phylogenies. Here, particular attention was given to the elucidation of the possible early origins of the eukaryotic lumen GTs. As reported previously [46], basic BLASTp searches did not detect prokaryotic homologues, so HMM profiles were constructed for each superfamily in order to look for them (see Methods [81]). The closest prokaryotic hits of each superfamily were used as seeds for psi-BLAST searches [82] against the bacterial and archaeal genomes. The vast majority of the detected prokaryotic sequences had PMT domains (for Protein *O-*Mannosylation Transferases) or the unknown protein domain DUF2029 defined from mycobacterial proteins (data not shown). The PMTs carry out protein *O-*mannosylation, which is the transfer of a mannose residue from Dol-P-Man to a protein in the lumen side of the ER membrane [83, 84]. *O-*mannosylation and *N-*glycosylation are highly intertwined, as inhibition of one enhances the other and both PMT and *N-*OST belong to the glycosyltransferase family C (GT-C) and may be distantly related [85]. *O*-mannosylation has been described in several bacteria including Mycobacteria [86, 87]–a suggestive result given the detection of the mycobacterial-based unknown domain DUF2029 in the distant lumen GT prokaryotic homologues. *O*-mannosylation has not been described in archaea yet, but it is exciting to point out that the protein Agl1, which was tentatively suggested to be a GT in *S. acidocaldarius* [88], bears a PMT domain. Incidentally, AglS is an archaeal protein which shares with the mannosyltransferases Alg3, Alg9 and Alg12 a Dol-P-mannose mannosyltransferase function [42]. AglS transfers a mannose to a S-layer protein *N-*glycan in *H. volcanii* and has also been suggested to be distantly related to the PMTs, but there is no obvious evolutionary relationship between AglS and the eukaryotic lumen GTs. Finally, the phylogeny of the PMT homologues was carried out (Additional file 7). The eukaryotic PMTs (including several paralogues) form a monophyletic clade (BPP = 1) but their relationship to their prokaryotic relatives is unclear (see Additional file 7 for details).

In summary, very distant sequence similarities were detected between the ER lumen GTs and the PMTs. PMTs use similar Dol-P-monosaccharide donors as the *N-*glycosylation lumen GTs (the same in the case of the mannosyltransferases), carry out similar transfer reactions and participate in functionally related mechanisms. As a result, the evolutionary relationship of these GTs with the PMT enzymes seems significant. It suggests that the closest ancestor of the lumen *N-*glycosyltransferases was not a sugar transporter [59] but a mannosyltransferase from the GT-C superfamily.

### Eukaryotic Stt3 was probably acquired from proteoarchaea

The *N-*OST that transfer the oligosaccharide from the lipid carrier to the acceptor protein have been genetically and biochemically described in eukaryotes [53, 89, 90], bacteria [44, 47] and archaea [91–95]. The evolution of the catalytic Stt3/AglB subunit of the *N-*OST is by far the best studied of the *N-*glycosylation enzymes. For instance, many HGTs and duplications of *aglB* have been reported in archaea and a close relationship between these proteins and eukaryotic Stt3 has been suggested [46, 47, 96, 97]. Yet, previous evolutionary studies were limited to one domain of life, were unable to determine the specific relationship of eukaryotes to archaea, or simply disregarded this issue.

**Fig. 6:**
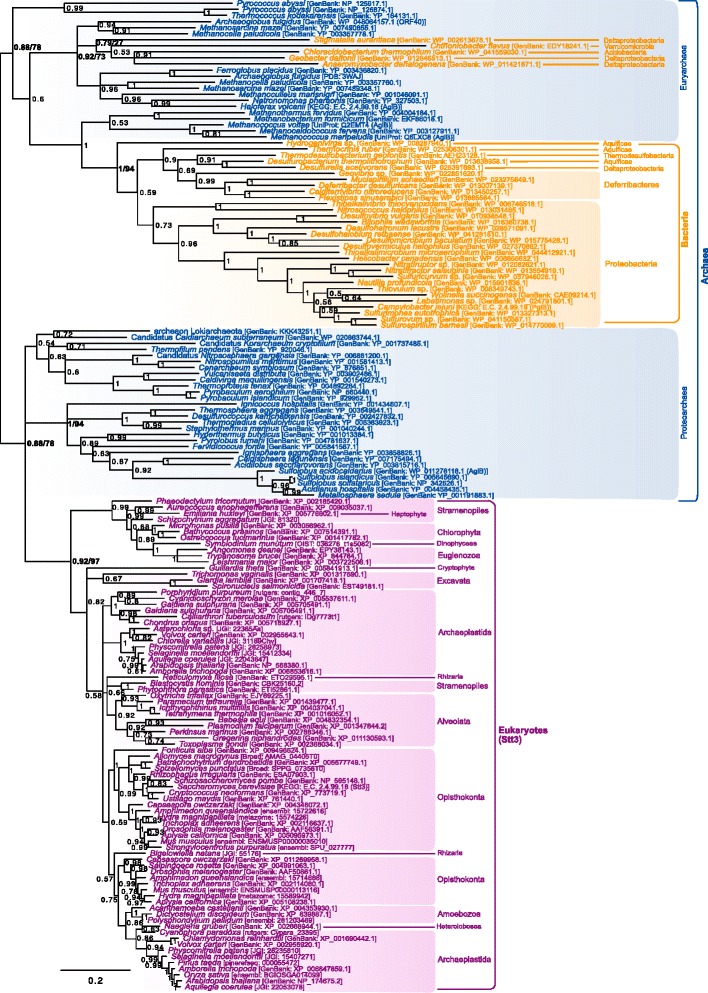
Bayesian phylogeny of the catalytic *N-*OST subunit (Stt3/AglB/PglB) homologues. The tree is unrooted and was reconstructed using 163 representative sequences and 268 conserved sites. Multifurcations correspond to branches with Bayesian posterior probabilities <0.5. Numbers at nodes indicate Bayesian posterior probabilities higher than 0.5. Bootstrap values from maximum likelihood analyses are reported on basal and major nodes. Colors on leaves represent the affiliation of sequences to a domain of life: archaea (*blue*), bacteria (*orange*) and eukaryotes (*purple*)

A phylogeny of Stt3/AglB homologues from all three domains of life was carried out (Fig. 6). The eukaryotic sequences form a monophyletic clade (BPP = 0.92) which contains clear taxonomic groups but whose phylogeny is blurred by the presence of several paralogues. The rest of the tree is made up of archaeal sequences roughly clustering according to the archaeal taxonomy and a few bacterial homologues located within the euryarchaeal clade. The widespread distribution of AglB homologues in archaea and the clustering of the sequences according to the main archaeal taxonomic groups supports the ancestral presence of this protein in LACA. Moreover, the location of the bacterial sequences within the euryarchaeal group supports the dominant opinion that this gene was transferred from archaea to bacteria [46–48] at least twice, followed by a few subsequent transfers among bacteria. The relationship between the archaeal and eukaryotic orthologues is more difficult to establish because the phylogeny is unrooted. Depending on where we decide to root this phylogeny, it may suggest that the gene was vertically inherited in archaea and eukaryotes from their last common ancestor (in a three-domain tree of life perspective), or that the eukaryotes branch within a paraphyletic proteoarchaeal group (BPP = 0.88, as shown in Fig. 6). This latter possibility is in agreement with the latest and most accurate phylogenies and phylogenomic studies, which support the closest relationship of the eukaryotic stem with the proteoarchaea [21–24]. Despite this result, no specific proteoarchaeal lineage, including *Candidatus* Lokiarchaeum [11], is specifically related to the eukaryotic Stt3, so the identity of the protearchaeal donor is unknown. This indicates a lack of resolution but it could also be explained by poor sampling among archaea or by the fact that the donor of this sequence predated LECA and, therefore, it cannot necessarily be assigned to one of the modern proteoarchaeal lineages.

Little is known about the function or evolution of the non-catalytic *N-*OST subunits [47]. Ost1, Ost2, Ost3/6, Swp1 and Wbp1 orthologues are widespread among eukaryotes (Table 1, Additional file 1), but homologues of the other subunits are more difficult to detect certainly because of their small size (e.g. yeast Ost4 is 36 amino acids long). BLASTp did not find any sequence similar to these subunits neither in bacteria nor in archaea, so distant prokaryotic homologues were looked for using HMM profiles. The few detected sequences participate in unrelated functions, so no promising candidates were found (data not shown). Maybe when more will be known about the functions of the non-catalytic *N-*OST subunits, it will be possible to make more informed choices to study the origin of these eukaryotic genes.

### A dolichol-P-monosaccharide synthase could have an archaeal origin

Although strictly speaking they are not components devoted to the *N-*glycosylation pathway, the two Dol-P-monosaccharide synthases supply mannose and glucose to the lumen GTs (Fig. 1b). These enzymes belong to the glycosyltransferase group 2 (GT2, [75]), which also comprises most GTs involved in the archaeal *N-*glycosylation pathways (Fig. 2). Therefore, the origins of the eukaryotic GT2 proteins were examined. In eukaryotes, Alg5 is a Dol-P-glucose synthase [98] and Dpm is a Dol-P-mannose synthase also providing mannose to GPI synthesis and *O*-mannosylation [99]. Two types of Dpm exist [75, 85, 100]: yeast Dpm has a C-terminal transmembrane helix to interact with membranes, whereas human Dpm1 lacks this structure and requires supplementary subunits (Dpm2/Dpm3). These differences were translated into two separate clades in previous Dpm phylogenies [46].

Similar to the GT1 superfamily, the GT2 superfamily comprises many proteins (red proteins in Fig. 2) that make its evolutionary study challenging. Only the closest prokaryotic relatives to the eukaryotic Alg5/Dpm sequences were kept for the analysis (see Methods). The resulting phylogeny is ambiguous (Additional file 8). The prokaryotic sequences are mixed, but the (unrooted) phylogeny seems to be split into two clades (BPP = 0.96): one dominated by bacteria and eukaryotic Alg5 sequences and another dominated by archaea and eukaryotic Dpm sequences. The bacterial-dominated clade contains WsfH from *P. alvei*, which may supply periplasmic Bac-P-Glc to tyrosine *O*-glycosylation [101]. This is remarkable because Alg5 are also Dol-P-Glc synthases, but it is balanced by the fact that AglD, a Dol-P-Man synthase from *H.volcanii,* is phylogenetically closer to the Alg5 clade*.* A few crenarchaeal sequences and a sequence from *Candidatus* Lokiarchaeum are basal to the eukaryotic Alg5 clade, but the significance of these sequences is uncertain because they are mixed to other euryarchaeal and bacterial sequences. Thus, the origin of the eukaryotic *alg5* gene is unclear.

The archaeal-dominated clade includes *H. volcanii* AglJ, *M. voltae* AglK, *A. fulgidus* Orf39 and the eukaryotic Dpm. As previously reported [46], the Dpm sequences are split into two groups depending on the presence of the C-terminal transmembrane helix. The eukaryotic Dpm clades are intertwined with several prokaryotic sequences, most notably the main proteoarchaeal clade of the tree. Thus, it is tempting to suggest that the eukaryotic sequences, as well as the bacterial sequences in this cluster, have proteoarchaeal origins. Since the Dpm1 type (devoid of a C-terminal transmembrane helix) is more widespread in eukaryotes, it is plausible that it was the ancestral type in eukaryotes and the fused type emerged later. The characterization of some proteoarchaeal homologues should allow testing this possibility.

In summary, the origins of the eukaryotic Alg5 are unclear, but Dpm may have a proteoarchaeal ancestor.

## Conclusion

*N-*glycosylation is a major protein modification in eukaryotes that modulates the properties of many proteins. Unraveling its origins is not only relevant to the particular case of this essential pathway, but its location in the ER membranes may also have implications for the origins of the ER and the eukaryotes themselves. Previous hypotheses suggested a particular evolutionary link between this eukaryotic pathway and archaeal *N-*glycosylation or one of the bacterial polyisoprenol-based pathways involved in cell wall synthesis [34, 48–50]. Yet, aside from a primordial paper that had neither the data nor the intention to tackle this issue [46], this is the first attempt to provide a thorough examination of the relationship of the eukaryotic *N-*glycosylation pathway with its prokaryotic relatives.

The proteins involved in polyisoprenol-based pathways from the three domains of life, including the eukaryotic *N-*glycosylation pathway, belong to a small number of gene families (Fig. 2). Only the putative *N-*glycosylation pathway from *S. acidocaldarius* has an obvious parallelism with the eukaryotic *N-*glycosylation, as they are both made up from representatives of the same protein superfamilies (Fig. 2). More detailed phylogenomic analyses, however, show that some eukaryotic *N-*glycosylation genes may indeed have proteoarchaeal ancestors, but only two of those belong to the *S. acidocaldarius* pathway. This stresses the fact that simple homology is not enough to draw robust evolutionary hypotheses about the relationship among polyisoprenol-based pathways. Not only the phylogenomic analyses are required to confirm evolutionary hypotheses, they also provide promising prokaryotic targets for the dynamic field of prokaryotic glycosylation characterization [36, 40].

**Fig. 7:**
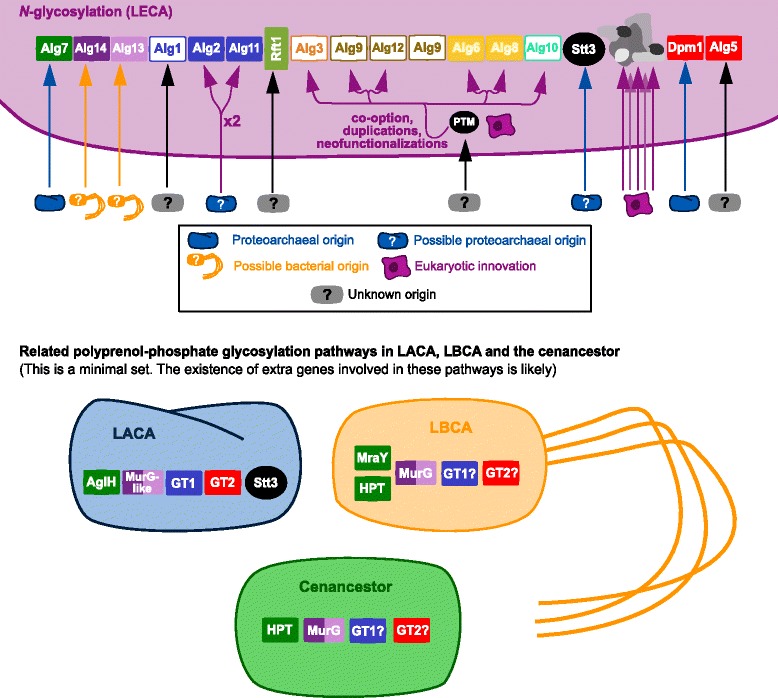
Origins of the eukaryotic *N*-glycosylation proteins and presence of related superfamilies in the last common ancestors of each domain of life and the cenancestor (summary)

Table 1 shows that most proteins involved in the eukaryotic *N-*glycosylation pathway are widespread in all major eukaryotic taxa. The phylogenetic results of each superfamily implicated in this process have been summarized in Fig. 7. Since many of these phylogenies are unrooted, their interpretation sometimes depends on the tree of life topology that we favor, but they clearly support a more complicated scenario for the origin of the eukaryotic *N-*glycosylation pathway than previously thought. All the canonical eukaryotic *N-*glycosylation genes were present in LECA and inherited (or lost/modified) in modern lineages, but the origins of the eukaryotic *N-*glycosylation genes are diverse. Alg7, Dpm1 and also possibly one GT1 and one Stt3 (*N-*OST catalytic subunit) have proteoarchaeal ancestors. A duplication of the original GT1 gene (blue family in Fig. 2) in the eukaryotic lineage before LECA would account for genes *alg2* and *alg11*. The *stt3* gene has been duplicated in some eukaryotic lineages too. The Alg13/Alg14 couple is structurally most similar to bacterial homologues, so it may have bacterial origins. The lumen GTs could have resulted from the co-option of a PMT protein involved in *O*-mannosylation and subsequent gene duplications. Finally, the other GT1 (Alg1), GT2 (Alg5) and the putative flippase have unknown origins and could be eukaryotic innovations. The origins inferred for the eukaryotic *N*-glycosylation genes will be open to reevaluation when more information will be available about their prokaryotic relatives that have been pointed out in this work.

Overall, this is another example that in metabolism, as in other functions, eukaryogenesis was achieved through an intricate combination of pre-existing and new elements. Although in a few cases (e.g. Alg13/Alg14, Alg2/Alg11, Stt3) a proto-eukaryotic origin cannot be completely ruled out, the results presented here suggest a strong influence of some proteoarchaeal genes in the making of the eukaryotic *N-*glycosylation pathway. This is in agreement with currently favored hypotheses which assume a proteoarchaeal ancestor to the eukaryotic stem [21–24]. For instance, these results are congruent with the “inside-out” hypothesis, which suggested that the presence of the eukaryotic *N-*glycosylation pathway in the ER membranes was a remnant of an eocyte S-layer *N-*glycosylation pathway ([14]). Yet, these results are also compatible with other eukaryogenesis hypotheses, as other eocyte or archaeal-stem hypotheses of eukaryogenesis may also account for the proteoarchaeal origin of these proteins [8, 10, 11] and the possibility that these genes were horizontally transferred from a proteoarchaeon to a non-proteoarchaeal eukaryotic stem cannot be excluded [3, 13, 15–18]. Whatever the eukaryogenesis hypothesis, this work shows that it is now time for works on eukaryotic origins to study the early evolution of the eukaryotic membranes and membrane functions.

## Methods

The original sequence seeds used to build the homology groups in Fig. 2 were selected from the literature (Table 2) and the KEGG database (http://www.genome.jp/kegg/). All vs. all reciprocal BLASTp searches were carried out [60]. The homology groups were manually built from the inspection of the reciprocal BLASTp: hits with an *e*-value inferior to 1e-05 were automatically considered to belong to the same homology group; proteins with two or more hits from the same homology group and *e*-values ranging between 0.01 and 1e-05 were also included in the homology group. Information from the literature was appealed to confirm or suggest particular homologies that were not unambiguously determined with simple reciprocal BLAST, e.g. in the case of the lumen GTs [59]. These cases of ambiguous homology appear as uncertain (empty white shapes in Fig. 2).

The same sequence seeds were used for the phylogenetic analyses. Local genomic databases were constructed for each domain of life from a selection of representative genomes (Additional file 9). BLASTp with default parameters were carried out against these local databases. These results were complemented with supplementary BLASTs against the same genomes carried out with alternative seeds and manual searches in the orthologue databases EggNOG (http://eggnogdb.embl.de/#/app/home, [102]) and OMA browser (http://omabrowser.org/oma/home/, [103]) to make sure that no orthologues were missed in the Table 1. Sequences too long, too short or for which the protein domain under study was not detectable using the Pfam database 28.0 (http://pfam.xfam.org/) were removed from the rest of the analyses. Some exceptions were applied when necessary as, for example, the euryarchaeal MurG-like homologues, which were kept for the N-terminal analysis despite their sequences being too divergent for Pfam to detect their respective protein domain in them. Preliminary alignments were carried out using MUSCLE v3.8.31 [104], sequences were trimmed using the program NET of the MUST package [105] and preliminary phylogenies were reconstructed using FastTree v.2.1.7 [106]. These preparatory analyses were examined for the overall topology of the trees, and used to select representative sequences for smaller datasets that could be used with more accurate phylogenetic reconstruction methods. Once the representative sequences had been selected, a new MUSCLE alignment was carried out on the Guidance server (http://guidance.tau.ac.il/ver2/, [107]). The alignments are available in the Additional file 10. The guidance statistical values on the alignment were used to make an informed choice to trim the sequences for the final phylogenetic reconstructions, and the trimmed alignments are reported in the Additional file 11. The final phylogenies were constructed using the programs MrBayes 3.2.6 [108] and RaxML-HPC2 8.1.24 [109] implemented in the CIPRES Science Gateway [110]. The MrBayes analysis used a LG substitution model [111] and 4 categories for the Γ distribution of substitution rates. Four chains of 1,000,000 generations were run, trees were sampled every 100 generations and the first 25 % generations were discarded as “burn in”. The RaxML reconstructions used the LG + Γ model [112] and 4 rate categories. A hundred bootstrap replicates were carried out with the same model to evaluate node support. The Bayesian and maximum likelihood phylogenies gave comparable results. The bootstrap support from RaxML of the main basal nodes was reported on the published Bayesian trees.

In some cases, the basic BLASTp search did not provide satisfactory results. When too many prokaryotic homologues were detected (e.g. GT1 and GT2 superfamilies), a smaller prokaryotic genome list was used for the preliminary analyses (Additional file 9). These earlier results were curated and only the closest homologues to the eukaryotic proteins of interest were kept. Supplementary sequences from the regular prokaryotic local genomic database were added later on to improve the prokaryotic diversity of the analyses. At that step, however, only the closest prokaryotic relatives were kept, so recent paralogues may have been missed. On the contrary, when very few similar sequences were detected in a domain of life, the searches were extended to the non-redundant (nr) sequences annotated as belonging to that domain of life on GenBank (http://www.ncbi.nlm.nih.gov/Genbank). If similar sequences could not be detected using these methods (e.g. lumen GTs in prokaryotes), HMM profiles were built from the MUSCLE alignments of the eukaryotic sequences and were used to look for distant homologues using the hmmsearch tool implemented in the HMMER v3.1b2 webserver (http://hmmer.org/, [81]). Homologues found in this way were validated as suitable candidates based on protein domain information from Pfam or functional annotation from the literature. The validated prokaryotic homologues were then used to carry out BLASTp or psi-BLAST searches [82], either against the regular list of genomes or the nr sequences from GenBank, depending on the results.

## Reviewers’ comments

The author thanks the reviewers for their comments. When possible, the manuscript has been modified according to their recommendations.

### First round of review

#### Referee 1, Pr. Patrick Forterre, University Paris Sud, France

##### Reviewer summary

It’s an interesting and useful paper that could be improved if fast evolving species were removed from the phylogenetic analyses.

## Reviewer comments

In this paper, Jonathan Lombard has performed a rather exhaustive phylogenomic analysis of N-glycosylation pathways in the three domains of life, focusing on the most conserved proteins, that belong to universally distributed protein glycosyltransferase superfamilies. This is an important work that could be useful for all scientists interested in reconstructing the evolution of membranes systems in the three domains. The paper is sometimes difficult to read for people outside of the fields because, for historical reasons, orthologous proteins have different names in different domains. Jonathan Lombard could benefit from his analysis to propose, when possible, new names, such that orthologous proteins in different domains could be readily identified.

Author’s response: *Thank you for this suggestion. I am aware of the fact that the number of proteins in this analysis is difficult to handle, especially because it is difficult to see the connection among homologues that carry out different functions in different lineages. Unfortunately, the field of polyisoprenol-related glycosylations is so vast that I have not been able to figure out a systematic notation that would make sense at the scale of the three domains. For instance, the HPT notation is already a generic name given to a superfamily that comprises proteins such as Alg7, AglH MraY, WecA, TagO,* etc. *When possible, I have referred to glycosyltransferase superfamilies (GT1, GT2,* etc.*), but in cases such as MurG and their homologues the renaming is difficult because of the complicated protein domain architecture of the family and the fact that many of these proteins are uncharacterized. I strongly recommend using the Fig.*2*as a general legend for the whole article, as it shows the relationships among all the proteins discussed here*.

Some of the proteins, such as HPT and MurG, indeed appear universal and the topology of their trees (Figs. 3 and 4) are rather consistent with those of classical universal proteins (Archaea and Eukarya closely related compared to Bacteria) (especially in the case of HPT). This suggests that these proteins are orthologous and were already present in LUCA as indicated by the author in Fig. 7. Could Jonathan Lombard speculate about the role of these proteins in LUCA? Can we conclude that LUCA already encoded glycosylated proteins at its surface? Is it possible to imagine a minimal glycosylation pathway that could have evolved into the complex pathways observed in Archaea/Eukarya and that was partly recruited in Bacteria for peptidoglycan biosynthesis?

Author’s response: *This article originally was longer and covered not only the origin of the eukaryotic N-glycosylation pathway but also the putative presence of a polyprenol-based glycosylation machinery in the cenancestor. In a previous submission, it was recommended to me to separate both subjects in order to make the messages clearer. As a result, I split the original article into this and another paper (reference* [50]*), where I discuss the implications for the last common ancestor of extant organisms (the cenancestor). In summary, the cenancestor most likely had a biosynthetic pathway that could have allowed the synthesis of a polyisoprenol-phosphate lipid carrier similar to Bac-P in this organism. Together with the presence of glycosyltransferases described in the present article, I conclude that a glycosylation machinery may have existed in the cenancestor. Since the overwhelming majority of the prokaryotic polyisoprenol-based glycosylation machineries are involved in the synthesis of cell wall components, I suggest that the cenancestor already had a mechanism to synthesize glycosylated cell walls. However, I could not unambiguously infer the presence of any oligosaccharide transferase (the proteins that ultimately select the final acceptor of the oligosaccharides) in the cenancestor, so I think that the system may have been less specific and developed into different cell walls glycosylation pathways later or just modified in the modern lineages because of the strong selection pressures that act on the glycocalyx. As this is the subject of another article, I have not included this discussion in this article, but I now refer to these possibilities at the end of the section “*The eukaryotic N-glycosylation initiation complex has a composite origin*”*.

Other proteins were probably present in the last common ancestor of Archaea and Eukarya or originated in Archaea if Eukarya emerge from within Archaea. Jonathan Lombard has interpreted all his results on the basis of the second hypothesis (the archaeal ancestor scenario) for the origin of Eukaryotes. However, this hypothesis is mainly supported by concatenation of unresolved single trees confused by the presence in the dataset of fast evolving species (Nanoarchaea, Methanopyrus, Korarchaeon) (see Forterre 2015). This is problematic since even the best phylogenetic methods cannot prevent long branch attraction in the presence of a very distant outgroup (Gouy et al., 2015), which is the case here with Bacteria. Better analyses in which most fast evolving species have been removed provided contradictory rootings. For instance, the analysis of Gribaldo and co-workers reject the TACK (proteoarchaeal) superphylum (Rayman et al. 2015) that the author favours here, grouping some Euryarchaea with “proteoarchaea”. The paper would be better if Jonathan Lombard consider in parallel the Lake (eocyte/proteoarchaea) scenario and the Woese’s scenario in which Archaea and Eukarya are sister groups forming a clade (Akarya, sensu Forterre, 2015). In that case, it is possible for instance that the Last Archaea and Eukarya Common Ancestor (LAECA) contained paralogues of some proteins, followed by differential loss in some archaeal phyla and in Eukarya. This could explain for instance why Eukarya are more related to particular archaeal phyla in the trees of Figs. 3, 5 and 6. Unfortunately, the phylogenie are difficult to interpret because Jonathan Lombard also uses many fast evolving species in his dataset. For instance, Nanoarchaeaum and the Korarchaeon in Fig. 3, Methanopyrus kandleri in Fig. 4. I would like to see new phylogenies without these fast evolving organisms. It’s not necessary to do time consuming Bayesian analyses considering the small number of positions analyzed. PhyML would be sufficiently informative.

Authors response: *New phylogenies were carried out removing the Nanoarchaeum, Korarchaea and Methanopyrus fast-evolving sequences. The Figs.*3*and*4b*have been replaced accordingly, but the other Figures of the paper did not contain sequences from these organisms or their impact was not obvious, so they were kept as they were. The overall topology of Fig.*3*did not change. Fig.*4b*changed in that, instead of branching within the bacterial EPS sequences, now the eukaryotic Alg13 homologues form a separate clade*.

*I agree on the fact that previous versions of this manuscript did not sufficiently discuss the phylogenies in the context of the traditional three domains of life topology. As these phylogenies are unrooted, a traditional proto-eukaryotic lineage cannot be ruled out from the interpretation of the Alg13/Alg14, Alg2/Alg11 and Stt3 phylogenies. I have now discussed these possibilities in the respective sections of the text. Yet, in order to provide as clear conclusions as possible in this already quite complicated article, I have decided to put forward the origins that I think are the most plausible in the abstract, final conclusions and the summary in Fig.*7*. This is based in particular functional and structural arguments discussed for each gene, but also in the fact that the rooting of eukaryotes within archaea is now favored by most authors (references* [19–22]*), including the work by Raymann et al. 2015 cited by the reviewer. I have also tried to forward in the conclusion the fact that the validity of these hypotheses may change when more information will be available about the prokaryotic relatives of the eukaryotic proteins studied here*.

I am also surprised by the small number of Thaumarchaeota in the trees. I did some rapid BLAST searches and much more sequences could be retrieved. Some sequences of Bathyarchaea, that usually branch with Caldiarchaeum and Thaumarchaea in protein trees could also be added to see if one recover the usual monophyly of these three groups that, in my opinion, should be all considered to be members of the phylum “Thaumarchaeota”. Surprisingly, the name Thaumarchaeota is never mentioned in this paper. This is damaging and can create some confusion. For instance in Fig. 5, the group “Proteoarchaea” includes only Crenarchaeal sequences and two Nitrososphera (Thaumarchaeota) that branch within Crenarchaeota (a probable gene transfer). This group thus correspond to Crenarchaeota and not to “Proteoarchaea”. This important information is lost when using the disputed phylum Proteoarchaea as an umbrella for diverse phyla. In general, I think dangerous and potentially misleading to talk about either the TACK or the Proteoarchaea superphyla. These superphyla are only valid if one root the archaeal tree between Euryarchaea and all other archaea, as suggested by Petitjean et al. (2014). These authors root the archaeal tree using Bacteria as outgroup. Using the same strategy, Rayman et al. (2015) find another root. It is in fact problematic to root the archaeal tree using such a distant outgroup. Using Eukarya as a much closer outgroup, Brochier-Armanet et al. rooted the tree between Thaumarchaea and other Archaea (Brochier-Armanet et al., 2008). We recently obtained the same rooting with a three domains topology of RNA polymerase after removing fast evolving sequences (manuscript in preparation) so the question is quite open.

Author’s response: *in addition to removing the fast-evolving sequences (see above), more thaumarchaea and Bathyarchaea sequences were also included in the new phylogenies presented in Figs.*3*and*4a*. This addition did not considerably change the topology of the resulting phylogenies, so the rest of phylogenies were kept unchanged. Regarding to the term “Proteoarchaea”, the annotations in the trees are meant to provide an indication of the taxonomic affiliation of the sequences used, and not at all to discuss the internal phylogeny of each domain of life. For instance, there are sequences in these figures that are put under the same grouping despite being paraphyletic, just to make the figures more readable. The names chosen are such as the number of annotations is both as few and precise as possible. For example, if a group contains mostly viridiplantae and a couple of rhodophytes, the whole group will be annotated as “archaeplastida”, whereas if it only contains chlorophytes, it will be annotated as such. According to the same principle, if a group of sequences is only made up of crenarchaea, it will be annotated as such, but if it also includes one thaumarchaea, it will be annotated as “proteoarchaea”, as this is the first higher taxonomic term that includes all sequences in the group (I prefer “proteoarchaea” to “TACK” as it is a name instead of an acronym and, therefore, there will always space for discussion of what is in the name). That being said, I do not think that the work by Raymann* et al. *2015 necessarily contradicts the proteoarchaeal monophily. That piece of work found that the euryarchaea were paraphyletic, with some of them forming a sister group to a monophyletic group of proteoarchaea. On the base of that work, the term that should be under scrutiny is “Euryarchaea”. The work by Brochier-Armanet* et al. *2008 showed that Cenarchaeum symbiosum (a Thaumarchaea) could be basal to all archaea when the eukaryotes were used as an outgroup, but the choice of that outgroup is questionable if the eukaryotes branch within the proteoarchaea, as some of the same authors suggest in the Raymann et al. 2015 paper. In short, although the monophyly of both the euryarchaea and proteoarchaea may be disputed, these groups are sufficiently well-established to be used in the annotation of the figures in this paper, which does not intend to solve the phylogeny of archaea anyway*.

A minor point; Jonathan Lombard use the term “Cenancestor” instead of LUCA for the Last Universal Common ancestor. However, the “Cenancestor” can be the common ancestor of any group. If the author wants to refer to the historical proposal by Fitch, he can consider that LUCA means the Last Universal CenAncestor!

Author’s response: *I have included the name of LUCA in the paper, as the reviewer is right in that this term is more generally known. Yet, the term “cenancestor” was kept throughout the text. The definition of this term is well stated in the paper, and I do not think that it is any more ambiguous than the “Universal” in LUCA*.

*Thank you for endorsing the publication of this work*.

Gouy R, Baurain D, Philippe H. Rooting the tree of life: the phylogenetic jury is still out. Philos Trans R Soc Lond B Biol Sci. 2015 370(1678):20140329. doi:10.1098/rstb.2014.0329.

Forterre P. The universal tree of life: an update. Front Microbiol. 2015 6:717. doi: 10.3389/fmicb.2015.00717.

Raymann K, Brochier-Armanet C, Gribaldo S. The two-domain tree of life is linked to a new root for the Archaea. Proc Natl Acad Sci U S A. 2015 112(21):6670–5.

Petitjean C, Deschamps P, López-García P, Moreira D. Rooting the domain archaea by phylogenomic analysis supports the foundation of the new kingdom Proteoarchaeota. Genome Biol Evol. 2014 7(1):191–204. doi: 10.1093/gbe/evu274.

### Referee 2, Dr. Sergei Mekhedov, NCBI, NLM, NIH, USA (nominated by Editorial Board member Michael Galperin)

#### Reviewer summary

I have no doubt that the paper by J. Lombard “The multiple evolutionary origins of the eukaryotic N-glycosylation pathway” deserves publication in Biology Direct. For the first time the author puts a phylogenetic analysis of N-glycosylation in the context of a metabolic pathway. He performs the analyses at large evolutionary distances comparing Eukaryotes, Archaea, and Bacteria and uses the state of the art methods. The main conclusions cannot be called unexpected but are nonetheless valuable.

## Reviewer comments

I have only a few critical remarks. 1. This is the first thorough phylogenetic study of the families involved. While phylogenies of most of the mentioned families have been studied at large distances in the study covering the majority of conserved protein families in Eukaryotes (Yutin et al., 2008), these phylogenetic trees have not been described or analyzed individually. The author presents a much more detailed study of the small number of families. Nevertheless, for a number of protein families he has to conclude that “the closest prokaryotic group to the eukaryotic clade is unclear”. In time with thousands new genomes sequenced and new methods of phylogenetic analyses developed this may change. Therefore, I strongly believe that the author or other researches will return to the study of these protein families in the future and at a very different level. Therefore, it might be helpful to add certain data to the Supplementary material of this paper. I would insist on publishing multiple alignments of all proteins involved, both trimmed and untrimmed. The last suggestion is prompted by the author’s argument that the topology of eukaryotic clade… “most likely results from the small number of alignment positions that was conserved in each phylogeny (119 and 97 respectfully)…” Trimming of the initial alignments was done by the author automatically and it happens that the trimming parameters used are the same for all the trees and therefore not optimal for some of them. Given the constantly changing and disappearing protein identifiers (and even gene models) in the public databases, it would be essential to provide stable identifiers, such as GenBank CDS IDs or UniProt\UniParc IDs.

Author’s response: *The untrimmed and trimmed alignments supporting all the phylogenies published in this article have now been made available in Additional files*9*and*10*[10 and 11 after second revision], respectively. The IDs provided in these phylogenies are updated NCBI’s accession numbers, as currently recommended in the NCBI’s website. Although the accession numbers may indeed change over time, it has always been possible tracing back old accession numbers to their new IDs. If, for some reason, the identifiers were to become completely inaccessible, the sequences will still be available on the Additional file*9*[10, after second review]*.

I failed to find word “ortholog” in the text. This is not a big deal, but I believe that the author should have at least checked the described protein families in some of the available databases of orthologous groups of proteins. I am convinced that his attempt to build his own homology groups by running BLAST all-against-all with an unjustified expectation value cut-off resulted in incomplete protein families for the list of Eukaryotes in Table 1. As an example, all question marks in Table 1 correspond to true orthologs of S. cerevisiae proteins which served as the basis for this table. The following proteins missed in Table 1 should be added.

- A. Missed orthologs of Ost4 (very short protein, present in many plants): >tr|Q5KCD6|Q5KCD6_CRYNJ Uncharacterized protein CNH00640 [Cryptococcus neoformans var. neoformans JEC21] > tr|I1FYU0|I1FYU0_AMPQE Uncharacterized protein [Amphimedon queenslandica] > tr|A0A0D2X2S9|A0A0D2X2S9_CAPO3 Uncharacterized protein [Capsaspora owczarzaki (strain ATCC 30864)] > tr|L8GRU2|L8GRU2_ACACA Uncharacterized protein [Acanthamoeba castellanii str. Neff] > sp|Q8L986|OST4B_ARATH Oligosaccaryltransferase [Arabidopsis thaliana] > tr|J3LPF6|J3LPF6_ORYBR Uncharacterized protein [Oryza brachyantha] > tr|A0A096R6U0|A0A096R6U0_MAIZE Uncharacterized protein [Zea mays] > tr|A0A059A7I3|A0A059A7I3_EUCGR Uncharacterized protein [Eucalyptus grandis] > tr|G7KZM4|G7KZM4_MEDTR Oligosaccaryltransferase [Medicago truncatula] > tr|W5B6B2|W5B6B2_WHEAT Uncharacterized protein [Triticum aestivum] > sp|C7J4U3|OST4A_ORYSJ Dolichyl-diphosphooligosaccharide--protein glycosyltransferase subunit 4A [Oryza sativa subsp. Japonica]
- B. Missed orthologs of Swp1 (dolichyl-diphosphooligosaccharide-protein glycotransferase, in most lineages orthologs are substantially longer than in S. cerevisiae) > tr|C5LE16|C5LE16_PERM5 Putative uncharacterized protein [Perkinsus marinus (strain ATCC 50983 / TXsc)] > tr|F2UPX0|F2UPX0_SALR5 Putative uncharacterized protein [Salpingoeca rosetta (strain ATCC 50818 / BSB-021)] > tr|B3SAA0|B3SAA0_TRIAD Putative uncharacterized protein [Trichoplax adhaerens] > tr|Q22ZG2|Q22ZG2_TETTS Oligosaccharyltransferase subunit ribophorin [Tetrahymena thermophila SB210] > tr|A0CJY2|A0CJY2_PARTE Uncharacterized protein [Paramecium tetraurelia] > tr|A0CN26|A0CN26_PARTE Uncharacterized protein [Paramecium tetraurelia] > tr|V9EKX7|V9EKX7_PHYPR Uncharacterized protein [Phytophthora parasitica P1569] > sp|Q5N7W3|RPN2_ORYSJ Dolichyl-diphosphooligosaccharide--protein glycosyltransferase subunit 2 [Oryza sativa subsp. Japonica] > tr|A9S283|A9S283_PHYPA Predicted protein [Physcomitrella patens subsp. Patens] > sp|Q93Z16|RPN2_ARATH dolichyl-diphosphooligosaccharide--protein glycosyltransferase subunit 2 [Arabidopsis thaliana] > sp|O74943|YJB6_SCHPO Uncharacterized protein C553.06 [Schizosaccharomyces pombe 972 h-] > tr|Q5KK05|Q5KK05_CRYNJ Uncharacterized protein CNC04720 [Cryptococcus neoformans var. neoformans JEC21] > tr|A0A015LNA6|A0A015LNA6_9GLOM Swp1p [Rhizophagus irregularis DAOM 197198w] > gi|46099108|gb|EAK84341.1| hypothetical protein UM03236.1 [Ustilago maydis 521] > tr|I1G5D9|I1G5D9_AMPQE Uncharacterized protein [Amphimedon queenslandica] > tr|Q7K110|Q7K110_DROME LD18774p [Drosophila melanogaster] > sp|Q9DBG6|RPN2_MOUSE Dolichyl-diphosphooligosaccharide--protein glycosyltransferase subunit 2 [Mus musculus] > tr|W4Y183|W4Y183_STRPU Uncharacterized protein [Strongylocentrotus purpuratus] > tr|A0A0D2U678|A0A0D2U678_CAPO3 Uncharacterized protein [Capsaspora owczarzaki (strain ATCC 30864)] > sp|Q54HG9|RPN2_DICDI Dolichyl-diphosphooligosaccharide--protein glycosyltransferase subunit swp1 [Dictyostelium discoideum] > tr|D3BAP4|D3BAP4_POLPA Dolichyl-diphosphooligosaccharide-protein glycotransferase [Polysphondylium pallidum] > tr|L8H4E5|L8H4E5_ACACA Ribophorin ii (Rpn2) protein [Acanthamoeba castellanii str. Neff] > tr|A8J449|A8J449_CHLRE Predicted protein [Chlamydomonas reinhardtii] > tr|D8TQJ2|D8TQJ2_VOLCA Putative uncharacterized protein [Volvox carteri] > tr|E1ZHV6|E1ZHV6_CHLVA Putative uncharacterized protein [Chlorella variabilis]
- C. Missed ortholog of Alg12 > tr|M1V5M0|M1V5M0_CYAM1 Uncharacterized protein CYME_CMM004C [Cyanidioschyzon merolae strain 10D]
- D. Missed ortholog of Alg14 > gi|146144751|gb|EAR99237.2| conserved hypothetical protein (macronuclear) [Tetrahymena thermophila SB210]

Author’s response: *The absence of the word “ortholog” is perfectly conscious. Orthologues are genes that have evolved through speciation and, in principle, they are mostly expected to carry out the same or very similar functions in different species. The proteins involved in eukaryotic N-glycosylation, however, belong to families with a high number of paralogues and probably xenologues, so I preferred using the broader homology rather than orthology throughout the article*.

*I think that there has been some kind of misunderstanding with regard to Table*1*and the formation of homology groups using all-vs-all BLAST, as both operations were completely independent from each other. On the one hand, Table*1*is a typical presence/absence table. It was made using the well-described Saccaromyces cerevisiae copy of each protein in the eukaryotic N-glycosylation pathway to look for homologues in all the eukaryotic genomes listed in the Additional file*8*[File 9 after second review]. I have included these sequences in the updated version of Table*1*, with the symbol “#”. I am grateful to the reviewer for pointing me towards these orthologues, that I had missed*.

*On the other hand, the all-vs-all BLAST was used only to build the homology groups shown in Fig.*2*. In order to do this, the sequences from proteins that had been described in various glycosylation pathways (Table*2*) were used to build a small database, and the all-vs-all BLAST was run only in that small database. The objective of that part of the study was to find out which proteins involved in polyisoprenol-based glycosylations were related to other proteins from other pathways. This provided a visual description of the relationship among the proteins in these pathways (Fig.*2*) and also determined the proteins that belonged to each superfamily, so they could be jointly taken into account in the superfamily phylogenies*.

*Thank you for endorsing the publication of this work*.

Reference Yutin, N., Makarova, K. S., Mekhedov, S. L., Wolf, Y. I., & Koonin, E. V. (2008). The Deep Archaeal Roots of Eukaryotes. Molecular Biology and Evolution, 25(8), 1619–1630.

### Second round of review

#### Referee 1, Pr. Patrick Forterre, University Paris Sud, France

The revised version has been quite improved. It was a very good idea to include both the untrimmed and trimmed alignments. Unfortunately, several nodes in the phylogenies are still not resolved after the removal of fast evolving species. This is probably because the small size of the proteins. It could be interesting to look in the alignment before trimming to look for indels supporting particular nodes.

Jonathan Lombard still favours the archaeal ancestor scenario for the origin of eukaryotes to explain the data because “most authors now favour an archaeal origin of the eukaryotic stem”. This is true but unfortunately, because this scenario is mostly based on very weak phylogenies (ref [8–11]) obtained from the concatenation of individual trees that are nor resolved and contained many fast evolving species (Ref [8–11]). There is only one 2010 reference indicating that it’s controversial (ref [18]). I would have added my two reviews in 2013 and 2015 in Archaea and Frontier in Microbiology in which biological arguments against the archaeal ancestor scenario and against published phylogenies are critically discussed.

Author’s response: *Thank you for your comments. Although there are phylogenomic works based on larger datasets and excluding fast-evolving sequences which also support the internal branching of the eukaryotes within archaea (*e.g. *Ref.* [24]*), I agree with Prof. Forterre on the fact that the origin of the eukaryotic stem is not set yet. Thus, I have integrated in the manuscript the articles suggested by Prof. Forterre. Despite this uncertainty about the eukaryotic origins, there are so many different genes in this pathway, each with a different story, that if I mentioned the implications for each gene of all different eukaryogenesis hypotheses in the conclusion, the message of the article would become quite confusing (for reminder, the main message of this article is that the eukaryotic N-glycosylation pathway had mixed origins). Yet, since I agree with the reviewer that the origin of eukaryotes is not set, I encourage the interested reader to refer to the “*Results and discussion*” section, where I develop other evolutionary explanations for each tree.*

Considering the nomenclature problem, Jonathan uses LACA, LECA, LBCA, but has still a problem with LUCA (also the name is now mentioned). I don’t understand why the U of LUCA is ambiguous? Universal, as in “universal proteins” clearly means all organisms encoding ribosomes since LUCA is the Last Common Ancestor of organisms from the three domains that have been defined based on ribosomal proteins (Forterre et al., 2014). Obviously, universal do not refer to the Universe. In any case, at the moment – and probably for quite a long time - we are only concerned in studying life on Earth. If life can be studied on planet X in the future, it will be easy to distinguish the terrestrial LUCA from the X LUCA! Beside, the “L” of LUCA is very important since all the ancestors of LUCA were also our Cenancestors. The term Cenancestor is therefore both ambiguous concerning the group of organism under consideration but also the identity of the ancestor among all ancestors of the group.

Author’s response: *I am very sorry, for my previous response was clearly insufficient. When I said that the word “universal” could also be considered somehow ambiguous, I was thinking to the fact that the term is very broad. Take the example of viruses: Prof. Forterre has been a prominent actor in the debate to determine if viruses are alive or not. I personally do not have a clear-cut opinion on the issue, but viruses clearly are a part of the “biological universe”. The same for prions and any other kind of molecular machines with various abilities. Was LUCA also the ancestor of some viruses? Maybe it was, but in the present work I only studied the genomes of extant cellular organisms, so I cannot say anything about a hypothetical ancestor of anything else.*

*This does not mean that I completely discard the term “universal” as being ambiguous: what I was trying to illustrate was the fact that any term can be ambiguous if not properly defined. And yet, in the present article the sense of the term “cenancestor” is fully defined as “the last common ancestor of extant organisms”. This definition includes the term “last”, which I agree is important, and identifies the group under consideration (extant organisms). The term “organism” itself could be considered to be ambiguous, but we need to draw the line somewhere and I think that the reference to “extant organisms” is quite clear of the fact that I refer to cellular life as we know it. I am not hostile to the term “LUCA”, though, and that is why I included it in the revised version of the manuscript. If I use the term of cenancestor it is a matter of preference, as the respective definitions of both terms are virtually identical*.

Jonathan concludes the presence of proteins used for the glycosylation of cell wall in LUCA. The term cell wall here is ambiguous since it often refers to the bacterial cell wall. In Archaea, glycosylation occurs at the level of the S-layer and in Eukaryotes at the level of the cytoplasmic membrane. This let open many options for the structure of the LUCA cell envelope.

Author’s response: *I have now made sure that the term “cell wall” is always qualified: “bacterial cell walls” refer to components of the bacterial cell structures beyond the plasma membrane (*e.g. *peptidoglycan, teichoic acids, exopolysaccharides,* etc.*); “archaeal cell walls” in principle refers to S-layer proteins, as relatively little is known about how the other archeal external cell structures are synthesized; “prokaryotic cell wall components” refer to both of the above; “cenancestral cell walls” are hypothetical, their nature is unknown and they are only mentioned because the implications of this work may infer their existence. Cell walls will be further discussed in the article to be published soon about the evolution of the polyisoprenol biosynthesis pathway. Eukaryotic cell walls are out of the scope of this article as they are synthesized in completely different ways, and they are never mentioned.*

Jonathan Lombard still does not use the name Thaumarchaea in the paper but still use in abundance the controversial term Proteoarchaea (a putative superphylum) that look like Proteobacteria (a phylum). I still have problem with this choice. There is a lack of logic. In several figure, group are indicated sometimes by the name of their superphylum and other time by the name of their phylum (for instance in Fig. 2*[sic, Figure 3?]*, crenarchaeota are sometime named proteoarchaea, sometimes crenarchae). In Fig. 4, thaumarchaea are not indicated despite the fact that they branch with Euryarchaea and not with crenarchaea.

Author’s response: *The objective of these labels is to show when the sequences in these trees group according to reasonable taxonomic clusters and when they do not. This is best done using a mix of names from superphyla, phyla or even names from lower taxonomic ranks (*e.g. *in eukaryotes)*.

*In Fig.*3*there is a group of sequences from crenarchaeota which is labeled as crenarchaea, and a group of crenarchaeota, thaumarchaeota, aigarchaeota, bathyarchaeota and lokiarchaeota which is annotated as “proteoarchaea”. In the latter case, 3 of the 5 suggested “phyla” only contain one sequence, because little genomic data is available for these groups. Yet, several works have suggested that all of these phyla may be closely related to each other and have been combined into a “superphylum” sometimes called TACK or proteoarchaea (Refs* [10, 53]*). In this particular case, the labeling as “proteoarchaea” is more informative, as it shows that the sequences group together according to known taxonomical clusters which would not be seen if each sequence was labeled with the name of the particular phyla of which sometimes they are the only representative. To take an apparently less controversial example, in the Additional file*2*most bacterial sequences are labeled with their phyla names, with the exceptions of the PVC (planctomycetes/verrucomicrobia/chlamydiae) supergroup and the Bacteroidetes/Chlorobi supergroup. But in other trees, when a group only contains planctomycetes or bacteroidetes, it is only labeled with the name of the phylum, not the superphylum. I do not think that there is a problem of logic there, since the objective of these labels is not to discuss what should be the definition of a microbial phylum or superphylum, but just to show that the phylogenetic clustering is taxonomically significant*.

*Regarding Fig.*4*, the original “proteoarchaeal” group was paraphyletic, so I changed the labeling completely. I hope that the new annotations will satisfy the concerns of the reviewer*.

Otherwise, it’s an important work that opens the way to future updated phylogenomic analyses of an important physiological pathway.

Author’s response: *thank you for your support, advice and interesting debates.*

Forterre P, Krupovic M, Prangishvili D. Cellular domains and viral lineages. Trends Microbiol. 2014, 22(10):554–558.

#### Referee 2, Dr. Sergei Mekhedov, NCBI, NLM, NIH, USA (nominated by Editorial Board member Michael Galperin)

I cannot understand why the author avoids not just using the word “ortholog” but also the analysis of orthologous relationships in the protein families involved in N-glycosylation. He writes in his response that there are many paralogs in these families. I disagree: these orthologous groups in Eukaryotes do not have many paralogs. Just look at Table 1! Most columns have only one symbol (+ or # or ?). Every one of these single symbols that I checked corresponds to a true ortholog of S.cerevisiae protein(s) used as the basis for Table 1. And if the author suspects that some are xenologs (which I could not identify having checked a part of Table 1) isn’t it the task of this publication to identify them?

Author's response: *I am sorry, in my response to the first review I did not realize that the reviewer was mainly concerned with the relationships among the eukaryotic sequences. I agree with Dr. Mekhedov on the fact that the eukaryotes sequences are most likely orthologues. The picture is less clear, though, when the evolutionary relationships are considered across the three domains of life. For instance, Fig.*2*shows multiple paralogues of the same families in prokaryotes, and some phylogenies support the existence of HGTs. Moreover, many of this prokaryotic homologues carry out completely different functions. In the new version of the manuscript, I have kept the term “homologue” to refer to comparisons among different domains of life and I now use the term “orthologue” to refer to the eukaryotic genes*.

I do not like the expression “distant/uncertain homology” in the legend of Table 1. “Uncertain” homologs, in my opinion, should not be published.

Author’s response: *I agree that the wording was not ideal. The relevance of these sequences was considered as uncertain because, despite being similar, they were extremely divergent, or short, or long or had any other specificity that would put into question the fact that they were actual orthologues carrying out the same function. Still, they are good candidates that it would be worth testing experimentally, so I think that they should be reported somehow. In the current version of the article I have been less conservative and I have removed that category (now all sequences appear with a “+” in Table*1*)*.

There are 23 columns in Table 1 each corresponding to a protein family involved in N-glycosylation. The paper provides analyses of five phylogenetic trees. Of course, the author might say that the other relevant trees are published in reference [50]. But it is not accessible. This may be appropriate in a draft, but not after final review. The author has either remove this reference or make sure that it is available.

Author’s response: *The objective of this paper was to look for the possible prokaryotic origins of the eukaryotic genes involved in the N-glycosylation pathway. There are 23 eukaryotic proteins considered in Table*1*, but only 10 of them have valuable homologues among prokaryotes. Owing to the fact that some of these proteins are paralogues of each other (Alg2/Alg11, Dpm/Alg5), the 7 phylogenies provided in this paper track the evolution of 9 of these 10 proteins. The remaining protein is Alg1, which is so divergent from the rest of the large GT1 superfamily that it was impossible to determine which were its closest prokaryotic relatives. A supplementary phylogeny is provided (the one of the PMTs), because of its possible relationship to the eukaryotic-specific lumen glycosyltransferases. From my point of view, these phylogenies cover all the proteins required to discuss the prokaryotic origins of the eukaryotic N-glycosylation pathway. There are no assumptions made in this article about the origins of the eukaryotic N-glycosylation pathway based on work published anywhere else*.

*There seems to be a confusion regarding the influence on the present article of an article currently under review (“Reference* [50]*” in the discussion with the reviewers). The present paper traces the origins of the eukaryotic N-glycosylation pathway, whereas the other studies the evolution of the polyisoprenol biosynthesis pathways (Dol-P & Bac-P) in the three domains of life. Both subjects are distantly related, but these are two independent pieces of work. The other article was mentioned earlier in relation to Prof. Forterre’s request to discuss possible glycosylation mechanisms in LUCA. But there is nothing in the present paper that directly relies on the other article. In order to avoid any misunderstanding, I have removed that reference and I now refer to it as follows: “The implications of these results [meaning the results in this manuscript] for the possible cenancestral cell walls will be discussed in a paper to be published soon (Lombard, data not shown).”*

The data presented in Table 1 are not complete and I can only guess why the author missed obvious paralogs. I would like to emphasize that the first step in phylogenetic analysis is collection of ALL relevant sequences. The author failed to do this. I do not think that it is referee’s job to find all missed proteins. I checked first three columns for missed paralogs. My general conclusion about why the author failed to collect ALL homologs: he used conventional unidirectional BLAST which simply does not detect all homologs. He does not give any cutoff values for BLAST. Unidirectional BLAST is not enough to identify all homologs in 2016! The author discarded the sequences that are too long which is also a significant mistake. He avoided this mistake in the case of fusions in Alg13 and Alg14 groups (because these fusions have been already documented in the literature), but why does he think that homologs in other families are always very similar in size? They are not! Neither are orthologs at large distances. This is the reason for his omission of MOST of the proteins in Swp1 group.

Obvious paralogs in Alg13 group (one of them missed in Table 1): gi|283436186|ref|NP_080523.2| putative bifunctional UDP-N-acetylglucosamine transferase and deubiquitinase ALG13 [Mus musculus]; gi|260166652|ref|NP_796104.2| glycosyltransferase 28 domain containing 1-like [Mus musculus]; gi|18405716|ref|NP_565950.1| UDP-GlcNAc:dolichol phosphate N-acetylglucosamine-1-phosphate transferase [Arabidopsis thaliana]; gi|15230258|ref|NP_191281.1| Glycosyl transferase family 4 protein [Arabidopsis thaliana].

Author’s response: *I now provide a completely revised version of Table*1*. This was made using my previous BLAST results and completed with supplementary BLASTs that were carried out using alternative seeds that in some cases revealed a few more eukaryotic orthologues that had been missed. Psi-BLASTs were also carried out but the results did not provide any additional information. Finally, the EggNOG and OMA databases were checked out for any other orthologue that I may have missed. The newly added Additional file*1*contains the accession numbers of all sequences that were reported in Table*1.

## Abbreviations

Bac-P, bactoprenol-phosphate; Dol-P, dolichol-phosphate; EPS, exopolysaccharide; GlcNAc, N-acetyl-glucosamine; GT, glycosyltranferase; GT1, glycosyltransferase superfamily 1; GT2, glycosyltransferase superfamily 2; HGT, horizontal gene transfer; HMM, hidden Markov model; HPT, polyprenol phosphate:N-acetylhexosamine-1-phosphate transferase superfamily; LACA, Last Archaeal Common Ancestor; LBCA, Last Bacterial Common Ancestor; LECA, Last Eukaryotic Common Ancestor; LPS, lipopolysaccharide; PMT, protein O-mannosyltransferase; TACK, Thaumarchaeota, Aigarchaeota, Crenarchaeota, Korarchaeota

## Additional files

Additional file 1: Accession numbers for sequences in Table 1. (XLS 63 kb) Additional file 2: Bayesian phylogeny of the HPT. (PDF 167 kb) Additional file 3: Full Bayesian phylogeny of the Alg13 and C-terminal catalytic domain of the MurG homologues. (PDF 139 kb) Additional file 4: Full Bayesian phylogeny of the Alg14 and N-terminal membrane domain of the MurG homologues. (PDF 139 kb) Additional file 5: Bayesian phylogeny of the Alg14 and N-terminal membrane domain of the MurG homologues, excluding bacterial MurG sequences. (PDF 134 kb) Additional file 6: Bayesian phylogeny of the Rft1 homologues. (PDF 213 kb) Additional file 7: Bayesian phylogeny of the PMT homologues. (PDF 139 kb) Additional file 8: Bayesian phylogeny of the closest relatives to the eukaryotic Alg5/Dpm homologues (GT2 superfamily). (PDF 181 kb) Additional file 9: List of the genomes used in this analysis. (PDF 106 kb) Additional file 10: Untrimmed alignments. (TXT 2684 kb) Additional file 11: Trimmed alignments. (TXT 397 kb)
