# Supplementary material for: The multiple evolutionary origins of the eukaryotic N-glycosylation pathway
Source: Biol Direct. 2016 Aug 4;11:36. doi: 10.1186/s13062-016-0137-2 (PMC4973528; doi:10.1186/s13062-016-0137-2)

Additional file 2. Bayesian phylogeny of the HPT. The tree is unrooted and reconstructed using 239 sequences and 171 conserved sites. Multifurcations correspond to branches with Bayesian posterior probabilities <0.5, whereas numbers at nodes indicate Bayesian posterior probabilities higher than 0.5. The bootstrap values from the maximum likelihood analyses have been reported on basal and major nodes. Colors on leaves represent the affiliation of sequences to their respective domain of life: archaea (blue), bacteria (orange) and eukaryotes (purple).

This phylogeny shows two large groups (BPP = 1), one mostly bacterial and the other containing most archaeal and eukaryotic sequences. The bacterial homologues are divided into an MraY group (BPP = 0.96) and another group containing enzymes involved in the synthesis of other bacterial cell wall components (e.g. LPS or teichoic acid, BPP = 0.95). Both bacterial subgroups show a wide bacterial diversity and most sequences cluster according to their taxonomic classification. This supports the ancestral presence of both paralogues in bacteria. A few plant sequences branch among the bacterial MraY as a sister group to the cyanobacteria. The expression of peptidoglycan synthesis genes is known to be required for plastid division in some plants [1], so the plastidial origin of these sequences is well supported. This bacterial clade also contains some divergent archaeal sequences that branch basally with regard to the miscellaneous bacterial paralogue (BPP = 0.76). However, their phylogenetic position should not be trusted because it was very sensitive to the alignment sites selected for the phylogenetic reconstruction (data not shown). A specific analysis of the archaeal/eukaryotic clade was carried out in Figure 1 of the main article.

1. Machida M, Takechi K, Sato H, Chung SJ, Kuroiwa H, Takio S, Seki M: **Genes for the peptidoglycan synthesis pathway are essential for chloroplast division in moss.** *Proc Natl Acad Sci USA* 2006, **103**:6753–6758.

Additional file 2

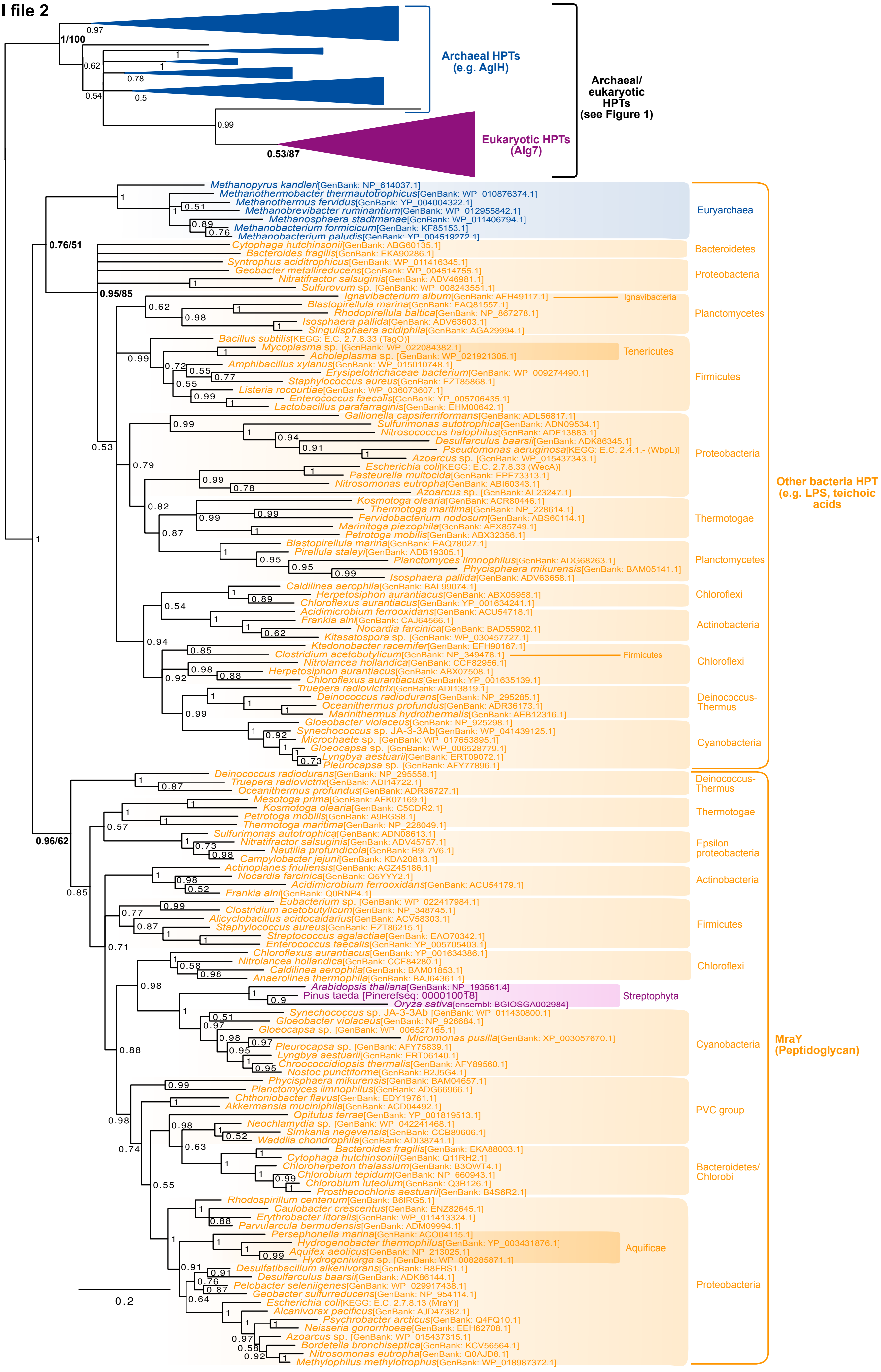

Supplement: Additional file 2: — Bayesian phylogeny of the HPT. (PDF 167 kb) [file 13062_2016_137_MOESM2_ESM.pdf]
