# Supplementary material for: The multiple evolutionary origins of the eukaryotic N-glycosylation pathway
Source: Biol Direct. 2016 Aug 4;11:36. doi: 10.1186/s13062-016-0137-2 (PMC4973528; doi:10.1186/s13062-016-0137-2)

Additional file 4. Full Bayesian phylogeny of the Alg14 and N-terminal membrane domain of the MurG homologues. The tree is unrooted and reconstructed using 189 sequences and 121 conserved sites. Multifurcations correspond to branches with Bayesian posterior probabilities <0.5, whereas numbers at nodes indicate Bayesian posterior probabilities higher than 0.5. The bootstrap values from the maximum likelihood analyses have been reported on basal and major nodes. Colors on leaves represent the affiliation of sequences to their respective domain of life: archaea (blue), bacteria (orange) and eukaryotes (purple).

In this phylogeny, a wide diversity of bacterial MurG sequences clustered within a separate clade (BPP = 0.99). The deep relationships among these bacterial sequences are unresolved, but most sequences group according to the main bacterial phyla, therefore supporting the ancestry of this protein in bacteria. The presence of this gene in the LBCA should not been surprising, as peptidoglycan synthesis is one of the most widespread cell wall biosynthesis in bacteria. The rest of the tree was made up of archaeal sequences, eukaryotic *N*-glycosylation proteins and mixed bacterial and archaeal sequences associated to the EPS functions. Since MurG proteins formed a clearly divergent group, they were removed and a more specific phylogeny was carried out to tackle the relationship of the eukaryotic sequences to their closest prokaryotic relatives (Additional file 4).

Additional file 4

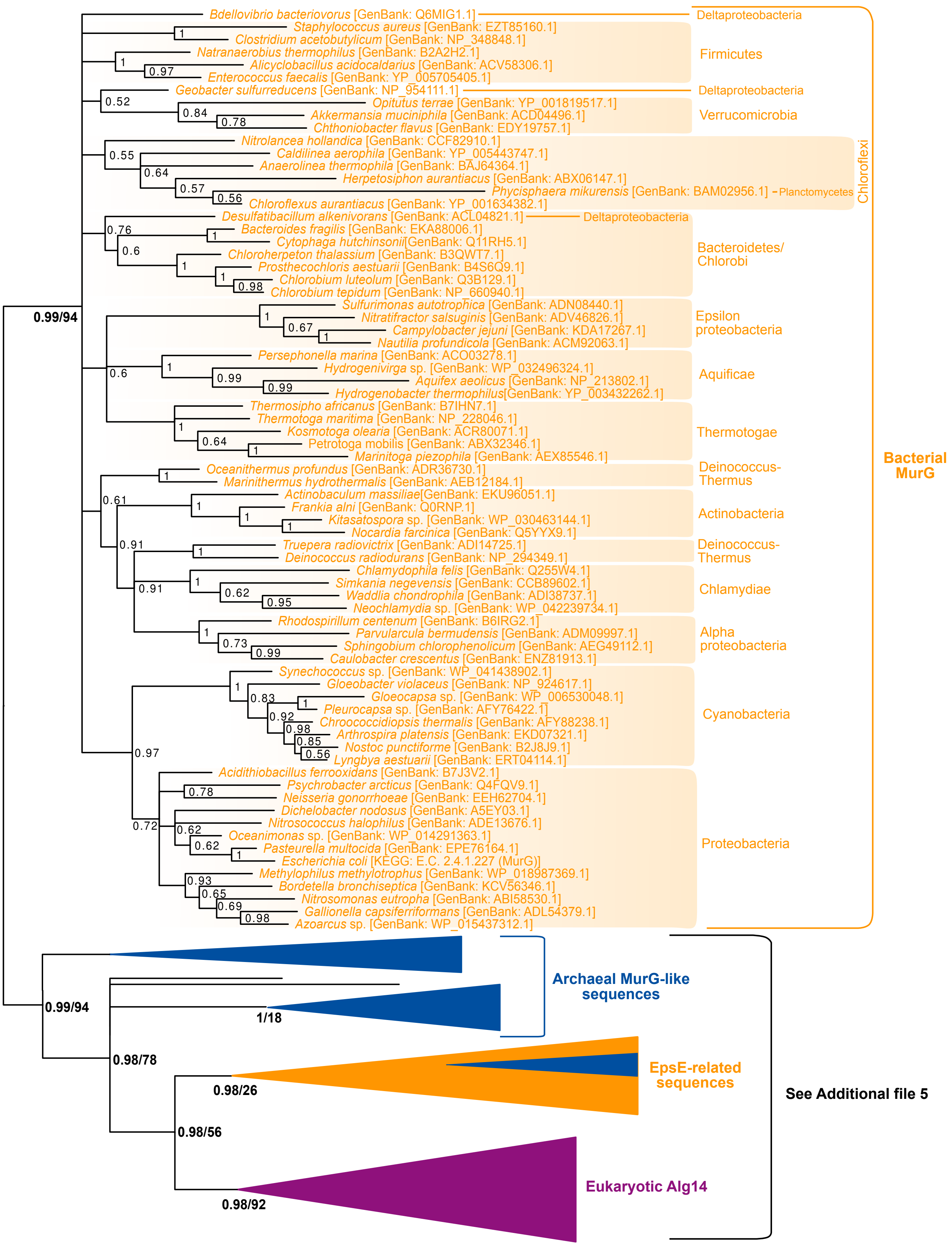

Supplement: Additional file 4: — Full Bayesian phylogeny of the Alg14 and N-terminal membrane domain of the MurG homologues. (PDF 139 kb) [file 13062_2016_137_MOESM4_ESM.pdf]
