# Supplementary material for: The multiple evolutionary origins of the eukaryotic N-glycosylation pathway
Source: Biol Direct. 2016 Aug 4;11:36. doi: 10.1186/s13062-016-0137-2 (PMC4973528; doi:10.1186/s13062-016-0137-2)

Additional file 5. Bayesian phylogeny of the Alg14 and N-terminal membrane domain of the MurG homologues, excluding bacterial MurG sequences. The tree is unrooted and reconstructed using 116 sequences and 97 conserved sites. Multifurcations correspond to branches with Bayesian posterior probabilities <0.5, whereas numbers at nodes indicate Bayesian posterior probabilities higher than 0.5. The bootstrap values from the maximum likelihood analyses have been reported on basal and major nodes. Colors on leaves represent the affiliation of sequences to their respective domain of life: archaea (blue), bacteria (orange) and eukaryotes (purple).

Additional file 5

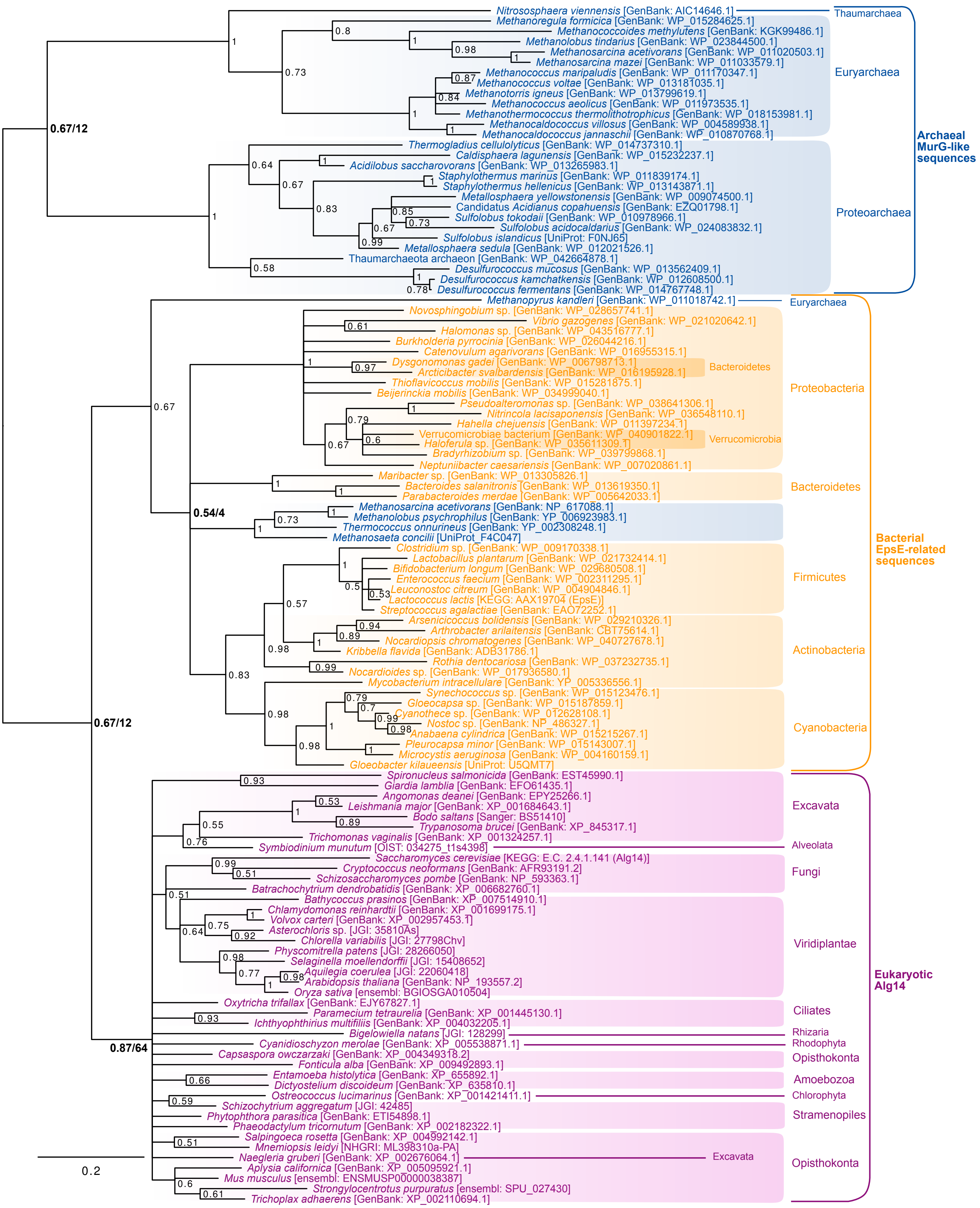

Supplement: Additional file 5: — Bayesian phylogeny of the Alg14 and N-terminal membrane domain of the MurG homologues, excluding bacterial MurG sequences. (PDF 134 kb) [file 13062_2016_137_MOESM5_ESM.pdf]
