# Supplementary material for: The multiple evolutionary origins of the eukaryotic N-glycosylation pathway
Source: Biol Direct. 2016 Aug 4;11:36. doi: 10.1186/s13062-016-0137-2 (PMC4973528; doi:10.1186/s13062-016-0137-2)

Additional file 6. Bayesian phylogeny of the Rft1 homologues. The tree is unrooted and reconstructed using 204 sequences and 274 conserved sites. Multifurcations correspond to branches with Bayesian posterior probabilities <0.5, whereas numbers at nodes indicate Bayesian posterior probabilities higher than 0.5. The bootstrap value from the maximum likelihood analyses has been reported on the eukaryotic clade. Colors on leaves represent the affiliation of sequences to their respective domain of life: archaea (blue), bacteria (orange) and eukaryotes (purple).

The identification of flippases has been traditionally difficult not only in *N*-glycosylation processes but also in some other polyisoprenol-related mechanisms such as peptidoglycan synthesis–the flipping process in LPS synthesis being much clearer [1–3]. In eukaryotes, the Rft1 protein was suggested to be the ER flippase in charge of translocating the initial heptasaccharide from the cytoplasm to the lumen side [4]. Although some involvement of Rft1 in eukaryotic *N*-glycosylation seems likely, its actual flippase activity has been called into question [5–7]. The very necessity of flippases is compromised by the fact that polyisoprenols are able to create hexagonal structures that attract proteins (maybe such as Rft1) to form membrane channels facilitating the translocation of the glycans across the membrane [8]. As a result, it might well be that the translocation is actually polyisoprenol-driven and the proteins are only accessory components to this mechanism.

Rft1 belongs to a family with homologues described in the three domains of life (Figure 2), of which the best characterized are those involved in bacterial EPS and *O*-antigen LPS syntheses [9–13]. Homologues of all the members of this family were searched in genomes from the three domains of life. The eukaryotic Rft1 homologues are monophyletic (BPP = 0.59), but the prokaryotic sequences are mixed both from a taxonomic and a functional point of view. This topology probably betrays frequent HGT, probably owing to the involvement of these proteins in transporting highly variable LPS or EPS components. This mixed tree may also result from difficulties in reconstructing the phylogeny of hydrophobic membrane proteins that conserve very little sequence similarity. In summary, this data does not allow us to clarify the origin of the eukaryotic sequences. Maybe when more structural information is known about these proteins, a more insightful evolutionary analysis will become possible.

1. Bouhss A, Trunkfield AE, Bugg TDH, Mengin-Lecreulx D: **The biosynthesis of peptidoglycan lipid-linked intermediates.** *FEMS Microbiol Rev* 2008, **32**:208–233.

2. Dell A, Galadari A, Sastre F, Hitchen P: **Similarities and differences in the glycosylation

mechanisms in prokaryotes and eukaryotes. *Int J Microbiol* 2010, **2010**:148178.

3. Guan Z, Naparstek S, Kaminski L, Konrad Z, Eichler J: **Distinct glycan-charged phosphodolichol carriers are required for the assembly of the pentasaccharide *N*-linked to the *Haloferax volcanii* S-layer glycoprotein.** *Mol Microbiol* 2010, **78**:1294–303.

4. Helenius J, Ng DTW, Marolda CL, Walter P, Valvano MA, Aebi M: **Translocation of lipid-linked oligosaccharides across the ER membrane requires Rft1 protein.** *Nature* 2002, **415**:447–450.

5. Frank CG, Sanyal S, Rush JS, Waechter CJ, Menon AK: **Does Rft1 flip an *N*-glycan lipid precursor?** *Nature* 2008, **454**:E3–4; discussion E4–5.

6. Rush JS, Gao N, Lehrman M a., Matveev S, Waechter CJ: **Suppression of Rft1 expression does not impair the transbilayer movement of Man5GlcNAc2-P-P-dolichol in sealed microsomes from yeast.** *J Biol Chem* 2009, **284**:19835–19842.

7. Jelk J, Gao N, Serricchio M, Signorell A, Schmidt RS, Bangs JD, Acosta-Serrano A, Lehrman M a, Bütikofer P, Menon AK: **Glycoprotein biosynthesis in a eukaryote lacking the membrane protein Rft1.** *J Biol Chem* 2013, **288**:20616–23.

8. Zhou G, Ii FAT: **NMR studies on how the binding complex of polyisoprenol recognition sequence peptides and polyisoprenols can modulate membrane structure**. *Curr Protein Pept Sci* 2005, **6**:399–411.

9. Feldman MF, Marolda CL, Monteiro A, Perry MB, Armando J, Valvano MA, Feldman MF, Marolda CL, Monteiro MA, Perry MB, Parodi AJ, Valvano MA: **The activity of a putative polyisoprenol-linked sugar translocase (Wzx) involved in *Escherichia coli O*-antigen assembly is independent of the chemical structure of the *O*-repeat.** *J Biol Chem* 1999, **274**:35129–35138.

10. Helenius J, Aebi M: **Transmembrane movement of dolichol linked carbohydrates during *N* -glycoprotein biosynthesis in the endoplasmic reticulum.** *Semin Cell Dev Biol* 2002, **13**:171–178.

11. Rick PD, Barr K, Sankaran K, Kajimura J, Rush JS, Waechter CJ: **Evidence that the *wzxE* gene of *Escherichia coli* K-12 encodes a protein involved in the transbilayer movement of a trisaccharide-lipid intermediate in the assembly of enterobacterial common antigen.** *J Biol Chem* 2003, **278**:16534–42.

12. Vorhölter F-J, Schneiker S, Goesmann A, Krause L, Bekel T, Kaiser O, Linke B, Patschkowski T, Rückert C, Schmid J, Sidhu VK, Sieber V, Tauch A, Watt SA, Weisshaar B, Becker A, Niehaus K, Pühler A: **The genome of *Xanthomonas campestris pv. campestris* B100 and its use for the reconstruction of metabolic pathways involved in xanthan biosynthesis.** *J Biotechnol* 2008, **134**:33–45.

13. Kaminski L, Guan Z, Abu-Qarn M, Konrad Z, Eichler J: **AglR is required for addition of the final mannose residue of the N-linked glycan decorating the *Haloferax volcanii* S-layer glycoprotein.** *Biochim Biophys Acta* 2012, **1820**:1664–1670.

Additional file 6

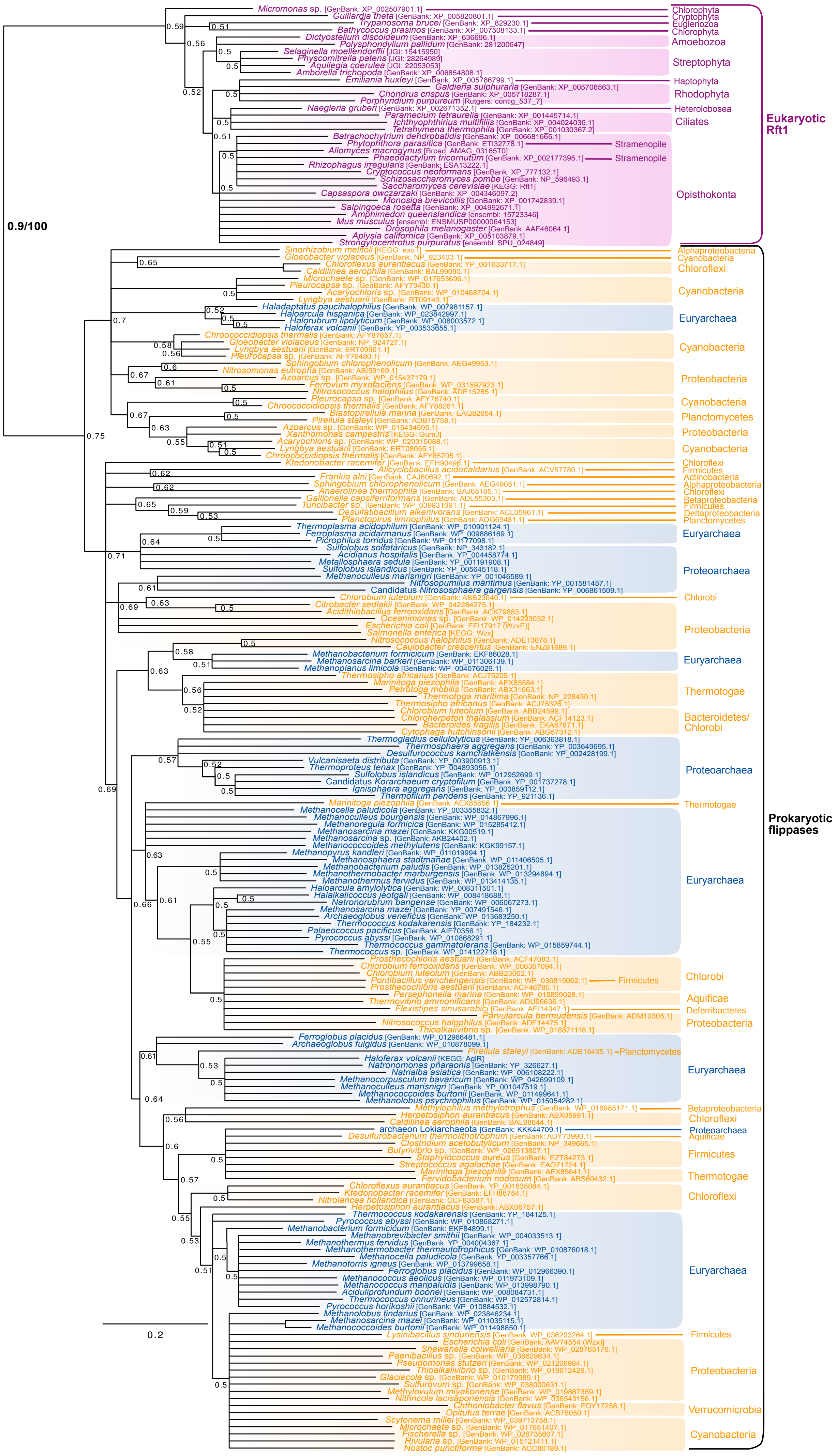

Supplement: Additional file 6: — Bayesian phylogeny of the Rft1 homologues. (PDF 213 kb) [file 13062_2016_137_MOESM6_ESM.pdf]
