# Supplementary material for: The multiple evolutionary origins of the eukaryotic N-glycosylation pathway
Source: Biol Direct. 2016 Aug 4;11:36. doi: 10.1186/s13062-016-0137-2 (PMC4973528; doi:10.1186/s13062-016-0137-2)

Additional file 7. Bayesian phylogeny of the PMT homologues. Tree is unrooted and reconstructed using 165 sequences and 119 conserved sites. Multifurcations correspond to branches with Bayesian posterior probabilities <0.5, whereas numbers at nodes indicate Bayesian posterior probabilities higher than 0.5. The bootstrap values from the maximum likelihood analyses have been reported on basal and major nodes. Colors on leaves represent the affiliation of sequences to their respective domain of life: archaea (blue), bacteria (orange) and eukaryotes (purple).

This phylogeny shows four main clades: one eukaryotic clade with a large number of known paralogues (BPP = 1), a proteoarchaeal clade (BPP = 1), a bacterial clade (BPP = 0.67) and a euryarchaeal clade (BPP = 0.78). In the long branch that separates the eukaryotic group from the main prokaryotic clades, several smaller bacterial groups are observed. In overall, the bacterial sequences do not show a clear separation into bacterial taxa across the tree, but the genes are widespread in a quite large diversity of organisms from this domain. The archaeal sequences display a better representation of the archaeal diversity. The clustering of archaeal sequences suggests that PMTs were ancestral at least in proteoarchaea and euryarchaea separately–and probably in LACA as well, in spite of the fact that the archaeal sequences are not monophyletic. The characterization of some of these prokaryotic sequences could help resolving the evolutionary connection of the eukaryotic proteins to their closest bacterial and proteoarchaeal relatives but, in the meanwhile, the origin of the eukaryotic PMTs remains unclear.

Additional file 7

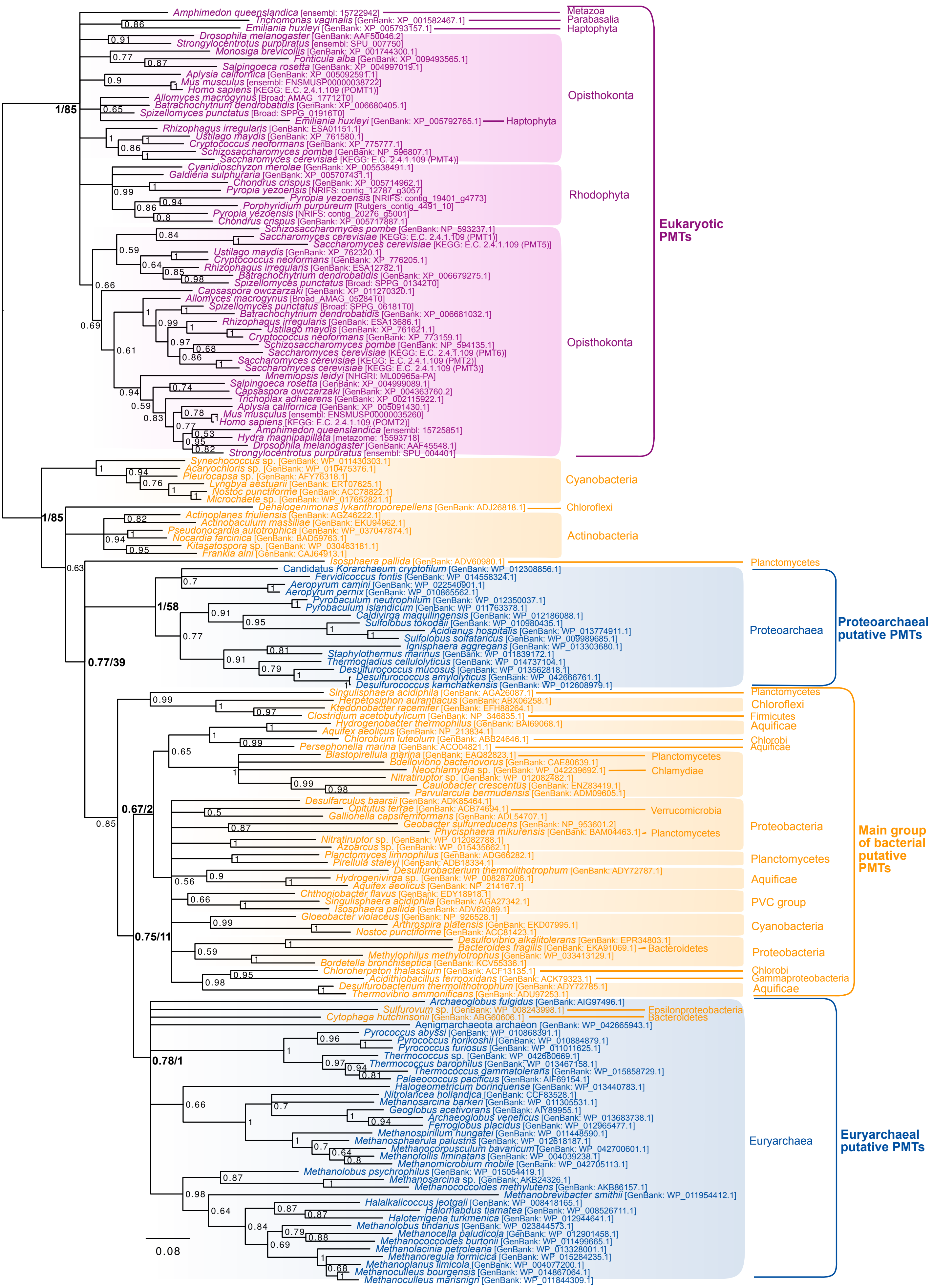

Supplement: Additional file 7: — Bayesian phylogeny of the PMT homologues. (PDF 139 kb) [file 13062_2016_137_MOESM7_ESM.pdf]
