# Supplementary material for: The multiple evolutionary origins of the eukaryotic N-glycosylation pathway
Source: Biol Direct. 2016 Aug 4;11:36. doi: 10.1186/s13062-016-0137-2 (PMC4973528; doi:10.1186/s13062-016-0137-2)

Additional file 8. Bayesian phylogeny of the closest relatives to the eukaryotic Alg5/Dpm homologues (GT2 superfamily). The tree is unrooted and was reconstructed using 247 representative sequences and 134 conserved sites. Multifurcations correspond to branches with Bayesian posterior probabilities <0.5, whereas numbers at nodes indicate Bayesian posterior probabilities higher than 0.5. The bootstrap values from the maximum likelihood analyses have been reported on basal and major nodes. Colors on leaves represent the affiliation of sequences to their respective domain of life: archaea (blue), bacteria (orange) and eukaryotes (purple).

Additional file 8

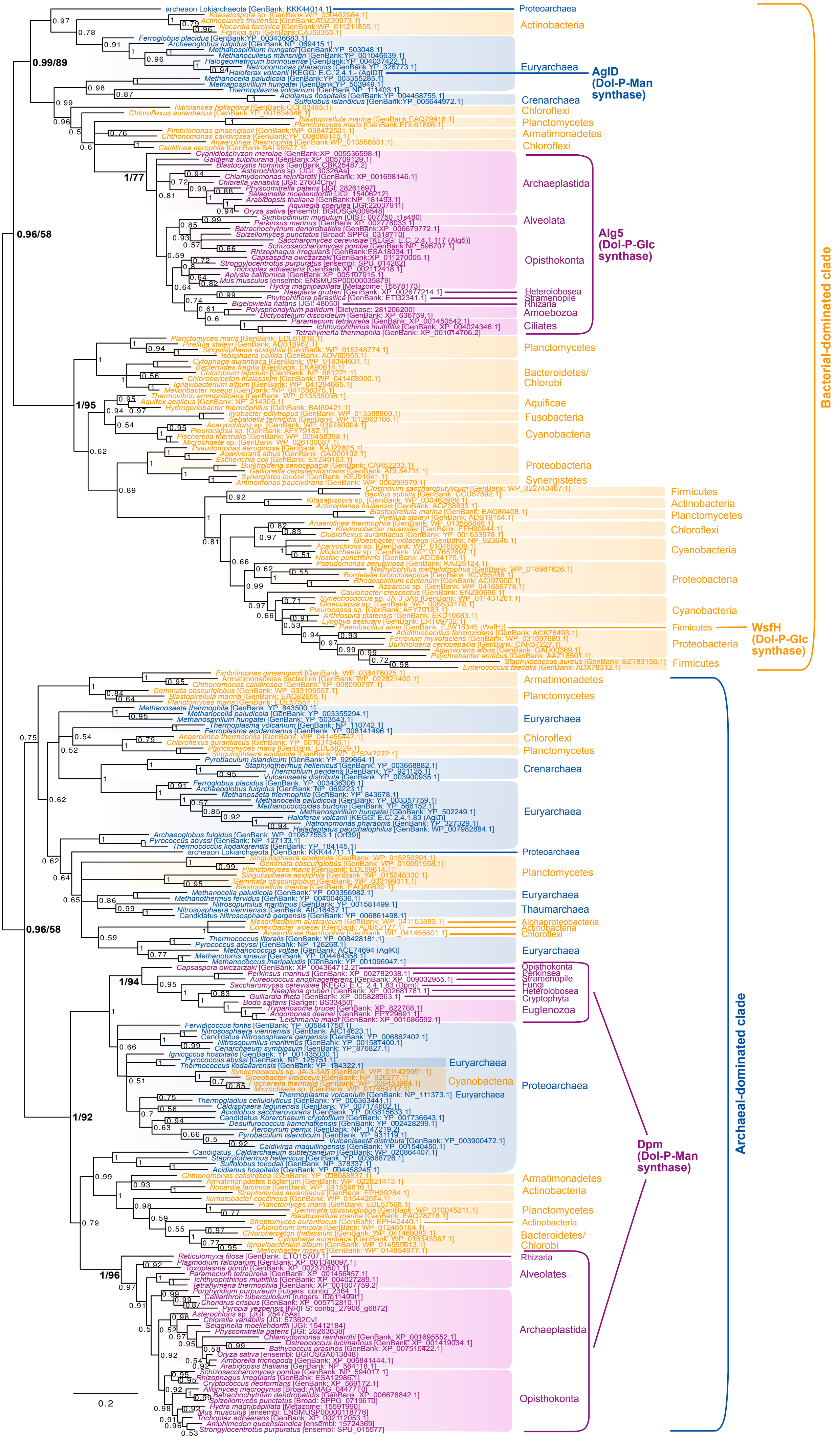

Supplement: Additional file 8: — Bayesian phylogeny of the closest relatives to the eukaryotic Alg5/Dpm homologues (GT2 superfamily). (PDF 181 kb) [file 13062_2016_137_MOESM8_ESM.pdf]
