# Supplementary material for: The multiple evolutionary origins of the eukaryotic N-glycosylation pathway
Source: Biol Direct. 2016 Aug 4;11:36. doi: 10.1186/s13062-016-0137-2 (PMC4973528; doi:10.1186/s13062-016-0137-2)
Supplement: Additional file 9: — List of the genomes used in this analysis. (PDF 106 kb) [file 13062_2016_137_MOESM9_ESM.pdf]

Additional file 9. List of the genomes used in this analysis. Asterisks indicate the shorter list of genomes that were used for some preliminary studies (GT1/GT2 superfamilies, see Methods).

Eukaryotes

*Acanthamoeba castellanii**
*Allomyces macrogynus**
*Amborella trichopoda**
*Amphimedon queenslandica**
*Angomonas deanei**
*Aplysia californica**
*Aquilegia coerulea**
*Arabidopsis thaliana**
Asterochloris sp. Cgr/DA1phos*
*Aureococcus anophagefferens**
*Babesia equi**
*Bathycoccus prasinos**
Batrachochytrium dendrobatidis JAM81*
*Bigelowiella natans**
*Blastocystis hominis**
*Bodo saltans**
*Calliarthron tuberculosum**
*Capsaspora owczarzaki**
*Chlamydomonas reinhardtii**
*Chlorella variabilis**
*Chondrus crispus**
*Cryptococcus neoformans**
*Cyanidioschyzon merolae**
*Cyanophora paradoxa**
*Dictyostelium discoideum**
*Drosophila melagonaster**
Emiliania huxleyi CCMP1516*
*Entamoeba histolytica**
*Fonticula alba**
*Galdieria sulphuraria**
*Giardia lamblia**
*Gregarina niphandrodes**
Guillardia theta CCMP2712*
*Hydra magnipapillata**
*Ichthyophthirius multifiliis**
*Klebsormidium flaccidium**
Leishmania major
Micromonas pusilla CCMP1545*
*Mnemiopsis leidyi**
*Monosiga brevicollis**
*Mus musculus**
*Naegleria gruberi**
*Oryza sativa**
*Ostreococcus lucimarinus**
*Oxytricha trifallax**
*Paramecium tetraurelia**
*Perkinsus marinus**
*Phaeodactylum tricornutum**
*Physcomitrella patens**
*Phytophthora parasitica**
*Pinus taeda**
*Plasmodium falciparum**
*Polysphondylium pallidum**
*Porphyridium purpureum**
*Prunus persica**
*Pyropia yezoensis**
*Reticulomyxa filosa**
Rhizophagus irregularis DAOM 181602*
*Saccharomyces cerevisiae**
*Salpingoeca rosetta**
Schizochytrium aggregatum ATCC 28209*
*Schizosaccharomyces pombe**
*Selaginella moellendorffii**
*Spironucleus salmonicida**
Spizellomyces punctatus DAOM BR117*
*Strongylocentrotus purpuratus**
*Symbiodinium minutum**
*Tetrahymena thermophila**
Thecamonas trahens ATCC 50062*
*Theileria annulata**
*Toxoplasma gondii**
Trichomonas vaginalis G3*
*Trichoplax adhaerens**
*Trypanosoma vivax**
*Ustilago maydis**
*Volvox carteri**

Archaea

Acidianus hospitalis W1*
Acidilobus saccharovorans 345-15
Aeropyrum pernix K1
Aigarchaeota archaeon JGI 0000001-A7

Archaeoglobus fulgidus DSM 4304*
Caldisphaera lagunensis DSM 15908
Caldivirga maquilingensis IC-167
Candidatus *Aenigmarchaeum subterraneum*
Candidatus *Caldiarchaeum subterraneum**
Candidatus *Haloredivivus* sp. G17
Candidatus *Iainarchaeum andersonii*
Candidatus *Korarchaeum cryptofilum* OPF8
Candidatus *Nanosalina* sp. J07AB43
Candidatus *Nitrososphaera gargensis* Ga9.2
Candidatus *Parvarchaeum acidiphilum* ARMAN-4_'5-way FS'
Candidatus *Parvarchaeum acidophilus* ARMAN-5_'5-way FS'
Cenarchaeum symbiosum A*
Desulfurococcus kamchatkensis 1221n
Ferroglobus placidus DSM 10642*
Ferroplasma acidarmanus fer1*
Fervidicoccus fontis Kam940
*Haladaptatus paucihalophilus**
Haloferax volcanii DS2
Halogeometricum borinquense DSM 11551*
Hyperthermus butylicus DSM 5456
Ignicoccus hospitalis KIN4/I*
Ignisphaera aggregans DSM 17230
Metallosphaera sedula DSM 5348*
Methanobacterium formicicum DSM 3637
Methanobrevibacter arboriphilus ANOR1
*Methanobrevibacter smithii**
Methanocaldococcus fervens AG86
*Methanocaldococcus jannaschii**
Methanocella paludicola SANAE*
*Methanococcoides burtonii**
Methanococcus maripaludis C5
Methanoculleus marisnigri JR1
Methanopyrus kandleri
*Methanosaeta thermophila**
Methanosarcina mazei Tuc01
*Methanospirillum hungatei**
*Methanotorris igneus**
Methanothermococcus thermolithotrophicus DSM 2095
Methanothermus fervidus DSM 2088*
Nanoarchaeum equitans Kin4-M
Natronomonas pharaonis DSM 2160*
Nitrosopumilus maritimus SCM1*
*Nitrososphaera viennensis**
Pyrobaculum aerophilum str. IM2
Pyrobaculum islandicum DSM 4184*
Pyrococcus abyssi GE5*
Pyrolobus fumarii 1A
*Staphylothermus hellenicus**
Staphylothermus marinus F1
Sulfolobus islandicus HVE10/4
Sulfolobus solfataricus P2
*Sulfolobus tokodaii**
Thermococcus kodakarensis KOD1
*Thermococcus litoralis**
Thermofilum pendens Hrk 5*
Thermogladius cellulolyticus 1633
Thermoplasma volcanium GSS1*
Thermoproteus tenax Kra 1
Thermosphaera aggregans DSM 11486
Vulcanisaeta distributa DSM 14429*

Bacteria

Acaryochloris sp. CCMEE 5410*
Acetobacter nitrogenifigens DSM 23921
Acholeplasma granularum ATCC 19168
Acidimicrobium ferrooxidans DSM 10331
Acidithiobacillus ferrooxidans ATCC 23270
Acidobacterium capsulatum ATCC 51196
Actinobaculum massiliae ACS-171-V-Col2
Actinoplanes friuliensis DSM 7358
Actinoplanes missouriensis 431
Actinopolyspora halophila DSM 43834
Aeromonas hydrophila 116
*Agarivorans albus**
Akkermansia muciniphila ATCC BAA-835
Alcanivorax pacificus W11-5
Alcanivorax sp. DG881
Algoriphagus marincola HL-49
Alicyclobacillus acidocaldarius DSM 446
Aminomonas paucivorans DSM 12260*
Ammonifex degensii KC4
Anaerolinea thermophila UNI-1*
Anaplasma phagocytophilum str. Dog2
Anoxybacillus kamchatkensis G10
Aquifex aeolicus VF5*
Armatimonadetes bacterium*
Arthrobacter aurescens TC1
Arthrospira platensis C1
Aurantimonas coralicida DSM 14790
Azoarcus sp. BH72

Bacillus subtilis B7-s*
Bacteroides fragilis HMW 610*
Bdellovibrio bacteriovorus HD100*
Bifidobacterium bifidum BGN4
Blastopirellula marina DSM 3645*
Bordetella avium 197N
Bordetella bronchiseptica 7E71
Borrelia burgdorferi 118a
Brachyspira innocens ATCC 29796*
Brevibacterium album DSM 18261
*Burkholderia cenocepacia**
Caldilinea aerophila DSM 14535 = NBRC 104270
Caldisericum exile AZM16c01
Calditerrivibrio nitroreducens DSM 19672
Caldithrix abyssi DSM 13497
*Campylobacter gracilis**
Campylobacter jejuni 10186
Candidatus *Aerophobus profundus*
Candidatus *Aminicenans sakinawicola* JGI OTU-1
Candidatus *Caldatribacterium californiense*
Candidatus *Calescibacterium nevadense* OTU 1
Candidatus *Fervidibacter sacchari* JGI OTU-1
Candidatus *Nitrospira defluvii*
Caulobacter crescentus NA1000*
Chlamydia pneumoniae B21
Chlamydophila felis Fe/C-56
*Chlorobium limicola**
Chlorobium luteolum DSM 273
Chlorobium tepidum TLS
Chloroflexus aurantiacus J-10-fl*
Chlorogloeopsis fritschii PCC 6912
Chloroherpeton thalassium ATCC 35110*
Chroococcidiopsis thermalis PCC 7203
Chrysiogenes arsenatis DSM 11915
Chthoniobacter flavus Ellin428
Chthonomonas calidirosea T49*
Clostridium acetobutylicum ATCC 824
Clostridium aerotolerans DSM 5434
*Clostridium saccharobutylicum**
*Collinsella tanakaei**
*Conexibacter woesei**
Coprobacillus sp. 3_3_56FAA*
Coprothermobacter proteolyticus DSM 5265
Corynebacterium diphtheriae 241
Cytophaga aurantiaca DSM 3654*
Cytophaga hutchinsonii ATCC 33406
Dehalococcoides mccartyi 195*
Dehalogenimonas lykanthroporepellens BL-DC-9
Deinococcus deserti VCD115
Deinococcus radiodurans R1*
Desulfarculus baarsii DSM 2075
Desulfatibacillum alkenivorans AK-01
Desulfobacter curvatus DSM 3379
Desulfovibrio alkalitolerans DSM 16529*
Desulfurispirillum indicum S5
Desulfurobacterium thermolithotrophum
*Desulfovibrio acrylicus**
Dethiosulfovibrio peptidovorans DSM 11002
Dichelobacter nodosus VCS1703A
Dictyoglomus thermophilum H-6-12
Elusimicrobium minutum Pei191
Enterococcus faecalis 62
Entomoplasma lucivorax ATCC 49196
Escherichia coli K2*
Ferrovum myxofaciens
Fervidobacterium nodosum Rt17-B1
Fibrobacter succinogenes S85
Fimbriimonas ginsengisoli Gsoil 348*
Fischerella muscicola PCC 7414
*Fischerella thermalis**
Flavobacterium antarcticum DSM 19726
Frankia alni ACN14a
Frankia sp. BMG5.23
*Fusobacterium nucleatum**
Fusobacterium periodonticum ATCC 33693
Gallionella capsiferriformans ES-2
Gemmata obscuriglobus UQM 2246*
Gemmatimonadetes bacterium KBS708
Gemmatimonas aurantiaca T-27
Geobacter sulfurreducens PCA
Geovibrio sp. L21-Ace-BES
Gloeobacter violaceus PCC 7421*
Gloeocapsa sp. PCC 73106
Glycomyces arizonensis DSM 44726
Halanaerobium praevalens DSM 2228
Halobacteroides halobius DSM 5150
*Halothermothrix orenii**
Helicobacter pylori 35A
Herpetosiphon aurantiacus DSM 785
Hydrogenivirga sp. 128-5-R1-1*
Hydrogenobacter thermophilus TK-6
Hydrogenobaculum sp. SN
Hyphomonas sp. 25B14_1
Ignavibacterium album JCM 16511*
*Ilumatobacter coccineus**
*Ilyobacter polytropus**
Isosphaera pallida ATCC 43644
Jiangella gansuensis DSM 44835

Kandleria vitulina DSM 20405
Kiloniella laminariae DSM 19542
Kineococcus radiotolerans ATCC BAA-149
Kitasatospora setae KM-6054
Kitasatospora sp. NRRL B-11411
Kordiimonas gwangyangensis JCM 12864
Kosmotoga olearia TBF 19.5.1*
Ktedonobacter racemifer DSM 44963*
Lactobacillus acidophilus ATCC 4796
Legionella pneumophila Leg01/16
Lentisphaera araneosa HTCC2155
Leptospira sp. B5-022
Leptospirillum sp. Group I
Leptotrichia buccalis C-1013-b
*Leptotrichia wadei**
Lyngbya aestuarii BL J
Marinimicrobia bacterium JGI 0000039-D08
Marinithermus hydrothermalis DSM 14884
Marinitoga piezophila KA3
Mariprofundus ferrooxydans PV-1
Meiothermus ruber H328*
Meiothermus silvanus DSM 9946
Melioribacter roseus P3M-2*
*Mesorhizobium australicum**
Mesotoga prima MesG1.Ag.4.2
Methylobacillus glycogenes JCM 2850
Methylophilus methylotrophus
Microchaete sp. PCC 7126*
Mitsuokella multacida DSM 20544*
Mycoplasma alkalescens 14918
Myxococcus xanthus DK 1622
Natranaerobius thermophilus JW/NM-WN-LF
Nautilia profundicola AmH*
Neisseria bacilliformis ATCC BAA-1200
Neisseria gonorrhoeae 1291
*Neisseria meningiditis**
Neochlamydia sp. S13
Nitratifractor salsuginis DSM 16511
Nitratiruptor sp. SB155-2
Nitrolancea hollandica Lb
Nitrosococcus halophilus Nc 4
Nitrosomonas eutropha C91*
Nitrospina gracilis 3/211
Nocardia farcinica IFM 10152*
Nocardiopsis alkaliphila YIM 80379
Nostoc punctiforme PCC 73102
Oceanimonas sp. GK1
Oceanithermus profundus DSM 14977
Opitutus terrae PB90-1
Parvularcula bermudensis HTCC2503
Pasteurella multocida 1500C
Pedobacter agri PB92
Persephonella marina EX-H1*
Petrotoga mobilis SJ95*
Phycisphaera mikurensis NBRC 102666*
Pirellula staleyi DSM 6068
Planctomyces limnophilus DSM 3776
*Planctomyces maris**
Pleurocapsa sp. PCC 7327*
Poribacteria bacterium WGA-3G
Prevotella aurantiaca JCM 15754
Propionibacterium acidipropionici DSM 4900
Prosthecochloris aestuarii DSM 271
*Pseudomonas aeruginosa**
Pseudomonas alcaliphila 34
Pseudonocardia autotrophica
Psychrobacter arcticus 273-4
Rhodopirellula baltica SH 1
Rhodospirillum centenum SW
Rickettsia felis URRWXCal2
Saccharopolyspora erythraea NRRL 2338
Salinimonas chungwhensis DSM 16280
*Sebaldella termitidis**
Simkania negevensis Z
Singulisphaera acidiphila DSM 18658*
Sorangium cellulosum So0157-2
Sphingobium chlorophenolicum L-1
*Sphingobium indicum**
Sphingobium sp. AP49
Spirochaeta sp.*
Spirochaeta thermophila DSM 6192
Staphylococcus aureus 1484-9
Streptococcus agalactiae 515
*Streptomyces aurantiacus**
Sulfurihydrogenibium subterraneum DSM 15120
Sulfurimonas autotrophica DSM 16294
Sulfurovum sp. AR
Synechococcus elongatus PCC 6301
Synechococcus sp. JA-3-3Ab*
*Synergistes jonesii**
Synergistes sp. 3_1_syn1
Terrimonas ferruginea DSM 30193
Thermodesulfatator atlanticus DSM 21156
Thermodesulfobacterium thermophilum DSM 1276
Thermodesulfovibrio islandicus DSM 12570
Thermomicrobium roseum DSM 5159
Thermosipho africanus TCF52B
Thermotoga maritima MSB8

*Thermotoga profunda**
Thermotoga sp. A7A
Thermovibrio ammonificans HB-1*
*Thermovirga lienii**
Thermus aquaticus Y51MC23
Thermus islandicus DSM 21543*
Thiobacillus thioparus DSM 505
Treponema bryantii NK4A124
*Treponema denticola**
Truepera radiovictrix DSM 17093*
Waddlia chondrophila WSU 86-1044
Xanthomonas oryzae ATCC 35933
Zavarzinella formosa DSM 19928
